# A root-based *N*-hydroxypipecolic acid standby circuit to direct immunity and growth of *Arabidopsis* shoots

**DOI:** 10.1038/s41477-025-02053-2

**Published:** 2025-07-22

**Authors:** Ping Xu, Sophia Fundneider, Birgit Lange, Rafał Maksym, Johannes Stuttmann, Anton R. Schäffner

**Affiliations:** 1 Institute of Biochemical Plant Pathology, Helmholtz Zentrum München, Neuherberg, Germany; 2 CEA, CNRS, BIAM, UMR7265, LEMiRE (Rhizosphère et Interactions Sol-Plante-Microbiote), Aix Marseille University, Saint-Paul lez Durance, France

**Keywords:** Microbe, Plant signalling, Plant molecular biology

## Abstract

Soil-borne microorganisms can systemically affect shoot resistance to pathogens relying on jasmonic acid and/or salicylic acid. However, the emanating root triggers in these scenarios remain elusive. Here we identify an *N*-hydroxypipecolic-acid-(NHP-)directed, salicylic-acid-related mechanism of root-triggered systemic resistance in *Arabidopsis*, which uses components of systemic acquired resistance known in leaves. However, in contrast to the inductive nature of systemic acquired resistance, FLAVIN-DEPENDENT MONOOXYGENASE 1 (FMO1) continuously synthesizes NHP in roots, while the glucosyltransferase UGT76B1 concomitantly conjugates and immobilizes NHP. Physical grafting experiments and tissue-specific knockouts revealed that the loss of UGT76B1 in roots leads to enhanced NHP release, initiating shoot responses. This counteracting standby FMO1/UGT76B1 circuit is specifically and sensitively modulated by root-associated microorganisms. Endophytic and (hemi)biotrophic fungi induce UGT76B1 degradation and FMO1 expression, resulting in varying levels of NHP being released to the shoot, where this root signal differently modulates defence and growth.

## Abstract (web summary)

Root microorganisms can trigger the release of immune-stimulating *N*-hydroxypipecolic acid from *Arabidopsis* roots by manipulating a standby circuit of its continuous synthesis and immobilization. *N*-hydroxypipecolic acid thereby influences shoot growth and pathogen defence.

## Main

In addition to local responses to pathogens, pests or chemical agents, defence against future challenges can also be triggered in distant tissues. These systemically spreading phenomena rely on various mechanisms and are broadly subsumed in ‘induced systemic resistance’ as advocated by De Kesel et al.^1–10^. Induced systemic resistance sensu stricto was originally coined to denote the enhanced shoot resistance triggered by the plant-growth-promoting rhizobacterium *Pseudomonas simiae* (former *fluorescens*) WCS417r, which was genetically dependent on jasmonic acid (JA) and ethylene (ET) responsiveness^1,11^. Numerous phenotypically similar instances of induced resistance and/or growth promotion were attributed to other root-associated microorganisms, such as *Fusarium*, *Colletotrichum* and *Trichoderma* species. Interestingly, the enhanced shoot defence status could also involve pipecolic acid and salicylic acid (SA) signalling in leaves, in conjunction or antagonistically with the JA/ET pathway^1,12–18^ (Supplementary Table 1). Furthermore, the impact of, for example, *Fusarium* and *Colletotrichum* strains on the root transcriptome has been assessed^14,19–23^ (Supplementary Table 1). However, the nature of the root signals triggering the shoot response remained elusive.

Systemic acquired resistance (SAR) is leaf-to-leaf induced systemic resistance enhancing the immune status in distant leaves upon a local infection. It is associated with enhanced expression of *PATHOGENESIS-RELATED* (*PR*) genes and largely depends on SA^10,24–26^. FLAVIN-DEPENDENT MONOOXYGENASE 1 (FMO1) is induced in the primary infected leaves, catalysing the biosynthesis of *N*-hydroxypipecolic acid (NHP). Among other long-distance signals, the movement of NHP is essential to trigger SA-dependent SAR in distant, systemic leaves^7,27–31^. The small-molecule glucosyltransferase UGT76B1 constitutes a negative regulator in this scenario. UGT76B1 is induced post infection to inactivate both NHP and SA via *O*-glucosylation and to attenuate defence. Accordingly, *fmo1* knockouts have compromised SAR^25^, whereas the loss of UGT76B1 leads to the autonomous activation of SAR^32–35^. It remains unclear whether SA-dependent shoot immunity triggered by root-associated microorganisms involves an analogous mechanism. Notably, UGT76B1 is constitutively expressed in the roots of naïve plants^36^, and, given that root uptake of NHP can potentially trigger shoot defence^37–39^, we reason that NHP could play a key role in mediating soil microbe–plant interactions and serve as a long-distance root-to-shoot signal.

## Results

### NHP is continuously synthesized and glucosylated in roots

**Fig. 1:**
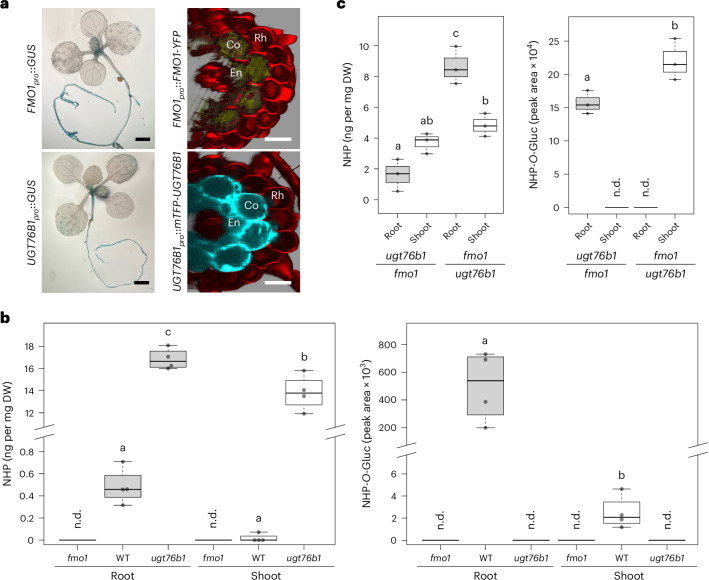
NHP biosynthesis and root–shoot mobility. **a**, Expression patterns of *UGT76B1* and *FMO1* transcripts and protein levels in 12-day-old naive plants grown on soil. Transcript levels were visualized via GUS staining using transgenic plants carrying *UGT76B1*_*pro*_::*GFP-GUS* and *FMO1*_*pro*_::*GUS* constructs (left). Protein levels were analysed via confocal laser scanning microscopy of main roots from two-week-old *ugt76b1* complemented with *UGT76B1*_*pro*_::*mTFP-UGT76B1* and *fmo1* complemented with *FMO1*_*pro*_::*FMO1-YFP* grown on half-strength Murashige and Skoog (MS) plates (right). Red indicates propidium iodide, yellow indicates FMO1–YFP and cyan indicates mTFP–UGT76B1. Scale bars, 3 mm (left) and 30 µm (right). En, endodermis; Co, cortex; Rh, rhizodermis. The experiment was repeated three times with similar results. **b**, Levels of NHP and NHP-*O*-Gluc in root and shoot tissues of two-week-old plants grown on half-strength MS plates, determined via LC–MS analysis. *n* = 4. Significant differences between roots and shoots of different genotypes were analysed using two-way analysis of variance (ANOVA) with post hoc Tukey’s test, as indicated by the letters (*P*_adj_ < 0.05). Overall, metabolite levels correlate with FMO1 and UGT76B1 transcript and protein levels. WT metabolites were analysed three times, and the additional reference values of *ugt76b1* and *fmo1* organs were obtained once. DW, dry weight; n.d., not detectable. **c**, NHP-deficient *fmo1* shoots were grafted onto *ugt76b1* roots (incapable of NHP glucosylation), and vice versa. Roots and shoots of three-week-old grafted plants were sprayed with 1 mM BTH and separately harvested for LC–MS analysis after two days. NHP-*O*-Gluc was detected exclusively in tissues containing a functional UGT76B1 enzyme, demonstrating its immobility. The distribution of NHP in rosettes and roots in both grafting combinations highlights NHP’s bidirectional mobility. *n* = 3. Root tissues are indicated in grey, shoot tissues in white. The boxes represent the interquartile range (IQR, Q1–Q3), with the median shown as a bold line. The whiskers extend to 1.5 × IQR. The experiment was conducted twice with similar results. Source data

The gene encoding the small-molecule glucosyltransferase UGT76B1 is induced in the shoot under stress conditions, where it glucosylates three defence-related compounds: isoleucic acid, SA and NHP^33,36,40^. However, its role in roots has remained unexplored, despite its constitutive expression in the root endodermis and cortex of naïve plants^36^. To explore this expression pattern, we analysed the co-regulation between *UGT76B1* and the genes involved in the biosynthesis of its substrates. We focused on genes involved in SA and NHP biosynthesis, since the processes leading to isoleucic acid are elusive. Among these, *FMO1* (encoding the final step of NHP biosynthesis) shows the highest co-expression with *UGT76B1* (Supplementary Table 2). Promoter–GUS experiments indicate a high basal expression of *FMO1* and *UGT76B1* in the root^36,41^ (Fig. 1a). For a more detailed examination, we used transgenic lines expressing fluorescent-protein-labelled UGT76B1 and FMO1. mTFP–UGT76B1 is present in both the endodermis and cortex, whereas FMO1–YFP^42^ is detectable only in the cortex (Fig. 1a). Additionally, root single-cell expression data largely confirm that *FMO1* is permanently expressed in the cortex and differentiating endodermis/cortex cells, whereas *UGT76B1* is strongly expressed in the endodermis, cortex and rhizodermis^43,44^ (Supplementary Fig. 1a,b). To detect products of both enzymes, we analysed extracts from roots and shoots using liquid chromatography–mass spectrometry (LC–MS). Consistent with the expression patterns, NHP and NHP-*O*-Gluc levels were notably higher in the roots than in the shoots of wild-type (WT) plants (Fig. 1b).

### NHP but not NHP-*O*-Gluc is bidirectionally mobile between root and shoot

Although NHP is known to move systemically between leaves^34,37^, its mobility between roots and shoots (as well as that of NHP-*O*-Gluc) remains unclear. To address this, reciprocal grafting was performed between *ugt76b1* and *fmo1*. Grafts between *ugt76b1*_shoot_ and *fmo1*_root_ enable NHP synthesis only in the shoot, with NHP *O*-glucosylation restricted to the root, and vice versa for *fmo1*_shoot_/*ugt76b1*_root_ plants. Grafted plants were treated with benzothiadiazole (BTH) to induce NHP biosynthesis, and roots and shoots were separately collected for metabolic analysis. NHP-*O*-Gluc was detectable only in tissues expressing UGT76B1, indicating its immobility. In contrast, NHP was found in both roots and shoots even in the absence of FMO1, strongly suggesting that NHP moves bidirectionally between roots and shoots (Fig. 1c).

### Lack of root expression of UGT76B1 induces shoot defence via NHP

**Fig. 2:**
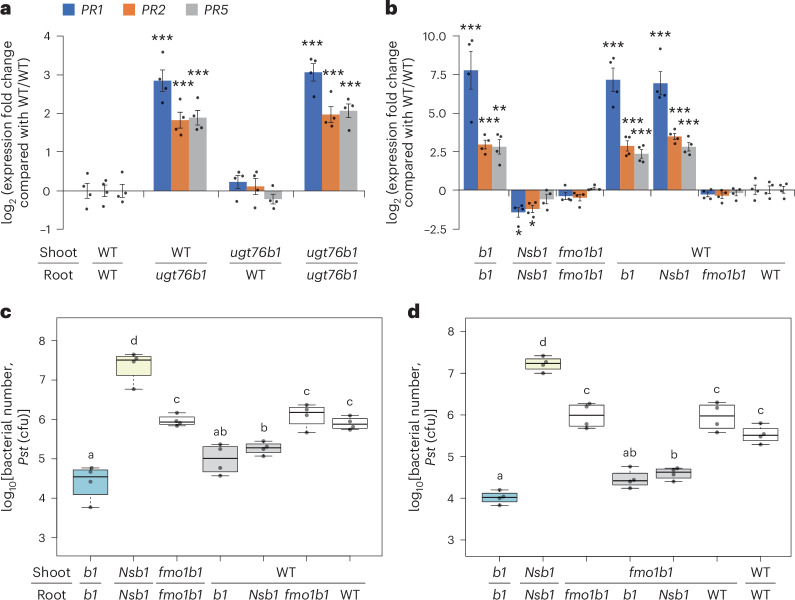
Impact of FMO1 and UGT76B1 root expression on shoot defence responses. **a**,**b**, Expression of SA-inducible defence genes *PR1*, *PR2* and *PR5* in shoots of grafted plants. The plants were grown under sterile conditions on half-strength MS plates. The bars represent means ± standard error of the mean; *n* = 4. The absence of UGT76B1 in roots is associated with the upregulation of *PR* genes in shoots, an effect not observed when *ugt76b1* is knocked out only in shoots (**a**). Root-*ugt76b1*-dependent enhancement of SA signalling marker expression in shoots is abolished by the additional loss of FMO1 in roots but is unaffected by SA depletion in roots through the introduction of bacterial SA hydroxylase NahG and SA biosynthesis gene *sid2* knockout (**b**). *b1*, *ugt76b1*; *Nsb1*, NahG *sid2* *ugt76b1*. Data from reverse transcription-quantitative PCR are normalized to the WT/WT combination; *S16* and *UBQ5* are used as reference genes. **c**, *ugt76b1* and NahG *sid2* *ugt76b1* homografts serve as extremely resistant and susceptible references, respectively (shown in blue and yellow), for comparison with WT and *fmo1* *ugt76b1* homografts. Enhanced defence against *Pst* DC3000 is observed when UGT76B1 is absent in roots. However, this enhancement is lost when FMO1 is also absent in roots, whereas the absence of root-expressed SID2 and the ectopic expression of the SA hydroxylase NahG do not impact this enhancement of defence. **d**, Similar to the grafting combinations in **c**, here *fmo1* *ugt76b1* was used as the shoot in heterografts due to its inability to produce or *O*-glucosylate NHP. The enhanced resistance in these shoots is exclusively dependent on root-synthesized NHP. *n* = 4. The boxes represent the IQR (Q1–Q3), with the median shown as a bold line. The whiskers extend to 1.5 × IQR. Significant differences between grafting combinations were analysed using the Welch two-sample *t*-test (*P < 0.05; **P < 0.01; ***P < 0.001) for **a** and **b**, or one-way ANOVA with post hoc Tukey test for **c** and **d** (indicated by letters, *P*_adj_ < 0.05). All experiments (**a**–**d**) were repeated twice with similar results. Source data

To investigate the role of UGT76B1 in the root, we generated localized knockouts in roots or shoots using grafts combining WT plants and *ugt76b1*. After a 16-day recovery, shoots of three-week-old plants were harvested for expression analysis. Compared with WT control homografts, shoots of *ugt76b1* homografts showed strong upregulation of the SA-inducible defence genes *PR1*, *PR2* and *PR5*. The local loss of UGT76B1 in roots also promoted the expression of these *PR* genes in WT shoots, whereas the loss of UGT76B1 in shoots alone did not affect their expression (Fig. 2a).

**Extended Data Fig. 1:**
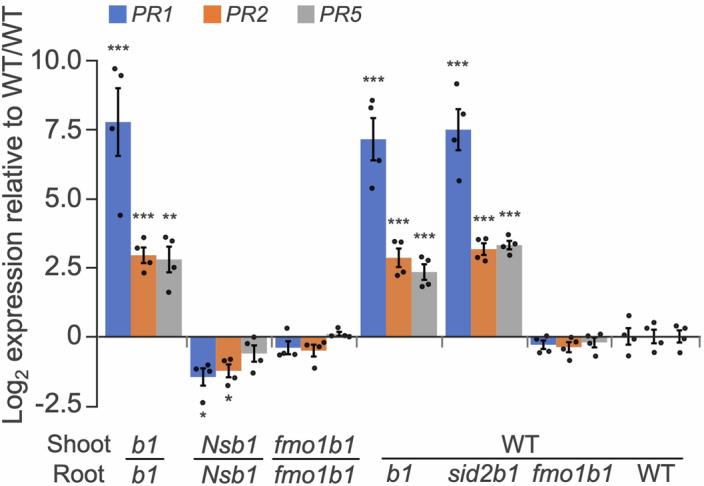
*ugt76b1* root-mediated shoot resistance is dependent on root *FMO1.* Since NahG may hydroxylate other benzoic metabolites, the *sid2 ugt76b1* double mutant was also used in grafting experiments, showing a similar effect to that of NahG *sid2 ugt76b1*. Bars represent means ± standard error of the mean, *n* = 4. Significant differences between genotypes were analyzed using the Welch two-sample t-test (*p < 0.05, **p < 0.01, ***p < 0.001). Experiments were independently repeated twice with similar results.

To assess whether UGT76B1 substrates in the root affect shoot phenotypes, we combined SA- and NHP-defective mutations with *ugt76b1*. In homografts, the induction of *PR* genes was abolished when *fmo1* or NahG *sid2* were introgressed into *ugt76b1*. However, in heterografts, *ugt76b1* root-induced *PR* gene expression in WT shoots was retained when SA was depleted in the root but abolished when NHP was depleted by the loss of FMO1 (Fig. 2b and Extended Data Fig. 1).

Similarly, *ugt76b1* roots enhanced the resistance of WT shoots against *Pseudomonas syringae* DC3000 (*Pst*), reaching the level of *ugt76b1* homografts. In WT_shoot_/ NahG *sid2* *ugt76b1*_root_ plants, the depletion of SA in roots did not alter the enhanced shoot resistance caused by the loss of UGT76B1 in roots, suggesting that root-derived SA does not influence shoot defence in this scenario. However, when FMO1 was eliminated in *ugt76b1* roots, the shoot resistance relapsed to WT levels (Fig. 2c). Due to the positive feedback loop of NHP biosynthesis^45^, root-derived NHP may amplify its biosynthesis in the shoot. Additional grafting combinations using *fmo1* *ugt76b1* as shoots were therefore tested for *Pst* resistance (Fig. 2d). As these shoots cannot synthesize or *O*-glucosylate NHP, their defence depends entirely on root-derived NHP. Consistently, root SA depletion did not impact the enhanced shoot defence induced by *ugt76b1* roots, whereas NHP depletion in roots abolished this effect. In conclusion, the enhanced defence of *ugt76b1* knockout plants results from NHP’s presence in roots, where it is constitutively synthesized and transported to the shoot in the absence of a counteracting glucosylation capability.

### UGT76B1 endodermal expression is critical for root-controlled shoot phenotypes

**Fig. 3:**
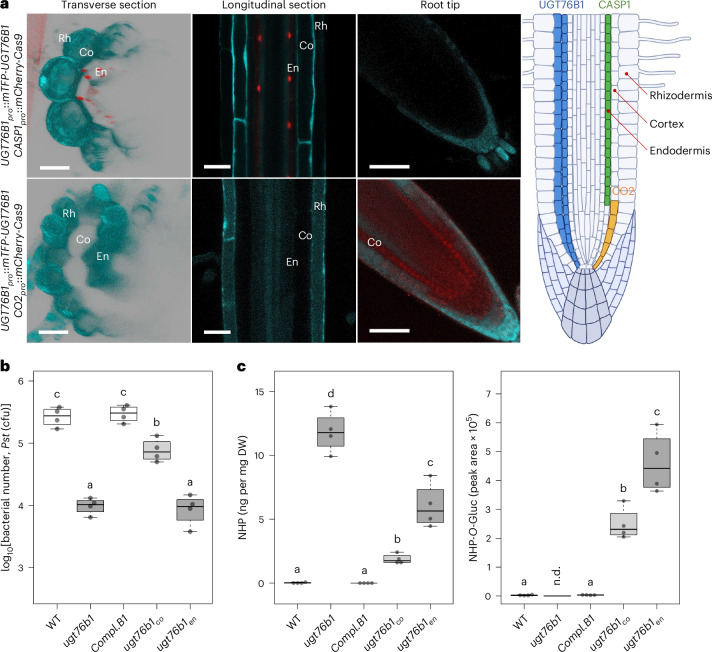
Differential impact of TSKO of UGT76B1 in root cell layers on shoot defence response. **a**, Confocal microscopy visualization of TSKO in 12-day-old plants grown on half-strength MS medium. The mTFP–UGT76B1 signal is shown in cyan, while mCherry–Cas9 driven by the *CASP1* and *CO2* promoters is detected in cortex initial and endodermal cells, respectively. The mTFP–UGT76B1 signal is abolished in tissues where mCherry–Cas9 is expressed from the *CASP1*_*pro*_::*mCherry-Cas9* and *CO2*_*pro*_::*mCherry-Cas9* constructs. *CO2*_*pro*_::*mCherry-Cas9* is expressed in cortex initial cells, efficiently knocking out the gene and resulting in the absence of the mTFP–UGT76B1 signal in differentiated cortex cells. Transverse sections were obtained via optical cross section from longitudinal *Z*-stacks. Scale bars, 30 µm. The experiment was repeated three times with similar results. **b**, Infection of four-week-old TSKO lines with *Pst* DC3000. The absence of UGT76B1 in the endodermis replicates the defence response observed in whole-plant knockouts, whereas its removal from the cortex layer results in moderately enhanced defence against the pathogen. The experiments were repeated four times. **c**, NHP and NHP-*O*-Gluc levels in the shoots of TSKO lines. *ugt76b1*_*en*_ and, to a lower extent, *ugt76b1*_*co*_ leaves contain enhanced NHP and NHP-*O*-Gluc levels compared with the WT. These measurements were performed once with *n* = 4 independent samples. The boxes (**b**,**c**) represent the IQR (Q1–Q3), with the median shown as a bold line. The whiskers extend to 1.5 × IQR. Significant differences between genotypes were analysed using one-way ANOVA with post hoc Tukey’s test, as indicated by the letters (*P*_adj_ < 0.05). Root section graphic in **a** created with BioRender.com. Source data

**Extended Data Fig. 2:**
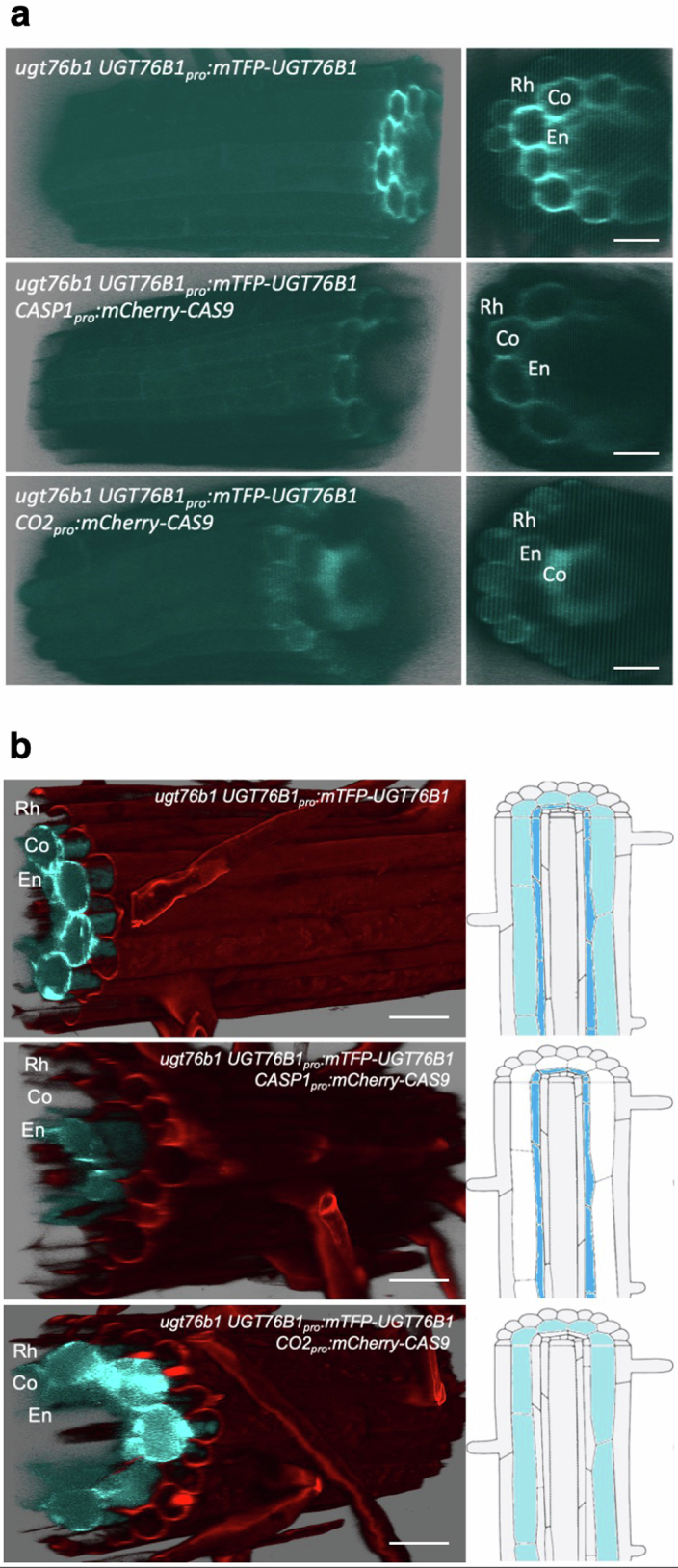
UGT76B1 expression in *ugt76b1* TSKO lines. Twelve-day-old *UGT76B1pro::mTFP-UGT76B1* and *ugt76b1*_*en*_ and *ugt76b1*_*co*_ TSKO plants grown on half-strength MS medium and examined by confocal microscopy to visualize the cellular expression pattern of mTFP-UGT76B1 and the efficacy of the TSKO approach. **a**, Efficacy of TSKO approach. Plants were treated with 1 mM BTH by surface spraying to induce (mTFP-)UGT76B1 expression beyond its level in naïve roots. The main root was examined by confocal microscopy two days post-treatment. The mTFP-UGT76B1 signal (cyan) was enhanced at the same localization as in naïve plants (see **b** and Fig. 3a), specifically the cortex and endodermis, with weak induction in the epidermis. In the *ugt76b1*_*en*_ TSKO line, the endodermis signal is not visible even after BTH induction. In the *ugt76b1*_*co*_ TSKO line, the cortex signal is absent, although induction is still observed in the rhizodermis. Bar, 30 µm. **b**, Illustration of three-dimensional UGT76B1 expression pattern in TSKO lines. Main roots of twelve-day-old plants grown on half-strength MS medium without BTH induction were used for confocal microscopy. mTFP-UGT76B1 is visualized in the cortex and endodermis. In the *ugt76b1*_*co*_ TSKO line, the mTFP signal is observed only in the endodermis. In the *ugt76b1*_*en*_ TSKO line, the endodermal signal is absent. En, endodermis; Co, cortex; Rh, rhizodermis. Bar, 30 µm. The experiments were repeated three times (**a**) or twice (**b**) with similar results.

To investigate the role of UGT76B1 at the cell-layer level, we employed tissue-specific knockout (TSKO) to achieve ‘genetic grafting’. We used a fluorescently labelled complementation line, *ugt76b1* *UGT76B1*_*pro*_::*mTFP-UGT76B1* (*Compl.B1*), as the parental line for TSKO, which allows visualizing and confirming the targeted knockout. An mCherry-labelled Cas9 protein was driven by the tissue-specific promoter *CO2* or *CASP1* to target *UGT76B1* in cortical or endodermal cells, respectively. In the endodermis-specific knockout line (*ugt76b1*_*en*_), the mTFP–UGT76B1 signal was absent in endodermal cells. In the cortex-specific knockout line (*ugt76b1*_*co*_), mTFP–UGT76B1 was eliminated in the cortex. Both knockouts were stable, with the targeted cell layers showing no mTFP signal (that is, UGT76B1 expression), even upon BTH induction of UGT76B1 (Fig. 3a and Extended Data Fig. 2).

**Extended Data Fig. 3:**
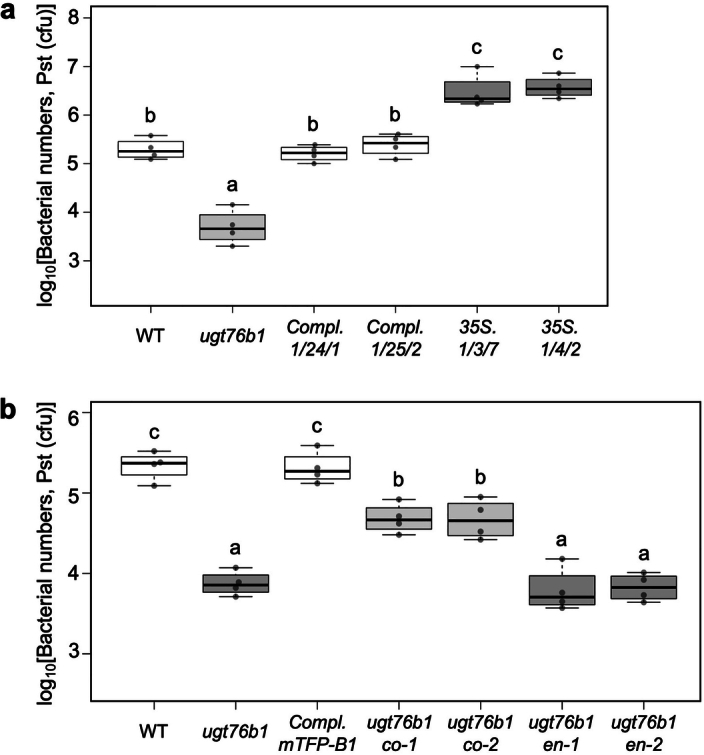
Independent complementation and TSKO lines. Sensitivity of independent complementation and TSKO lines towards infection with *Pst* DC3000. **a**, Two complementation lines (1/24/1 and 1/25/2), in which *ugt76b1* was complemented with the *UGT76B1*_*pro*_*::mTFP-UGT76B1* construct. Line 1/25/2 was selected as the parental line for TSKO and used as a reference, named *Compl.mTFP-B1*. Additionally, two overexpression lines (1/3/7 and 1/4/2) carrying *35S*_*pro*_*::mTFP-UGT76B1* were tested; line 1/4/2 was selected for further confocal microscopy analysis. **b**, The absence of UGT76B1 in the endodermis replicates the enhanced defense status observed in whole-plant knockouts, whereas its removal from the cortex layer results in a moderately enhanced defense against the pathogen. Two independent cortex- and endodermis-specific knockout lines were obtained and tested for *Pst* proliferation. *ugt76b1co-1* and *ugt76b1en-2* were selected for repeated disease assays and microscopy analysis. The experiments (**a, b**) were independently repeated twice with similar results. Boxes represent the interquartile range (Q1–Q3), with the median shown as a bold line. Whiskers extend to 1.5 × IQR, *n* = 4. Significant differences between genotypes were analyzed using one-way ANOVA with post-hoc Tukey test, as indicated by letters (*p*_adj_ < 0.05). Source data

**Extended Data Fig. 4:**
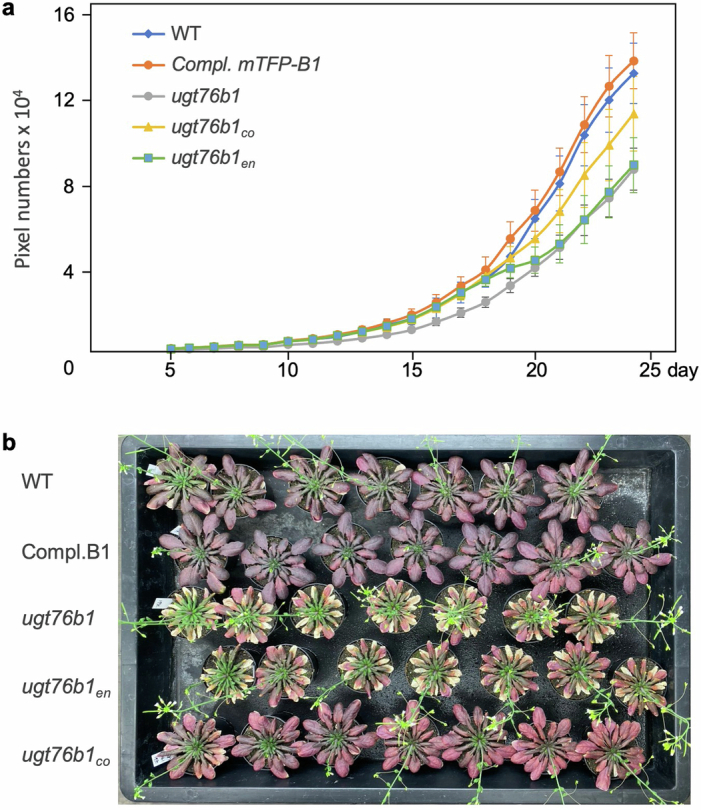
Growth and leaf phenotypes. **a**, Growth monitoring of TSKO lines. Data collection began from 5-day-old plants; leaf projection area as a proxy for rosette growth was determined by the region with active chlorophyll fluorescence, measured daily at 6 a.m. (Extended Data Fig. 9a for a correlation of projected leaf area and fresh weight biomass). WT and the complementation line (*Compl.B1*) exhibit similar growth. *ugt76b1*_*en*_ shows a growth pattern similar to the *ugt76b1* mutant, whereas the growth of *ugt76b1*_*co*_ falls between that of WT and the *ugt76b1* mutant. *n* = 12, error bars represent standard deviation. **b**, Anthocyanin accumulation and early senescence induced by day length shift. Four-week-old plants grown under short-day conditions were shifted to long-day conditions for an additional week. *ugt76b1* and *ugt76b1*_*en*_ exhibited similar early senescence, yellowing phenotypes, with less visible purple color in the leaves, indicating reduced anthocyanin accumulation. *ugt76b1*_*co*_ leaves displayed a visually lighter purple color compared to WT and the complementation line. Both experiments (**a, b**) were independently repeated twice with similar results. Source data

Upon *Pst* infection, *Compl*.*B1* exhibited susceptibility similar to that of the WT, indicating successful complementation. The enhanced resistance of *ugt76b1*_*en*_ against *Pst* matched that of the full-knockout mutant, while *ugt76b1*_*co*_ exhibited intermediate resistance between the WT and *ugt76b1* (Fig. 3b and Extended Data Fig. 3). This enhanced resistance is consistent with the elevated NHP levels in both TSKO lines. However, unlike *ugt76b1*, both TSKO lines accumulated high levels of NHP-*O*-Gluc, as they retain intact UGT76B1 in the shoot (Fig. 3c). Additionally, *ugt76b1* plants showed slower growth, earlier senescence^36^ and lower anthocyanin accumulation than the WT. *ugt76b1*_*en*_ mirrored these phenotypes, whereas *ugt76b1*_*co*_ was intermediate between the WT and *ugt76b1* (Extended Data Fig. 4a,b). These results suggest that endodermal expression of UGT76B1 is critical, as its absence in this specific root cell layer replicates the full-knockout phenotype.

### UGT76B1 and FMO1 distinctly and specifically react to different types of soil microorganisms

**Fig. 4:**
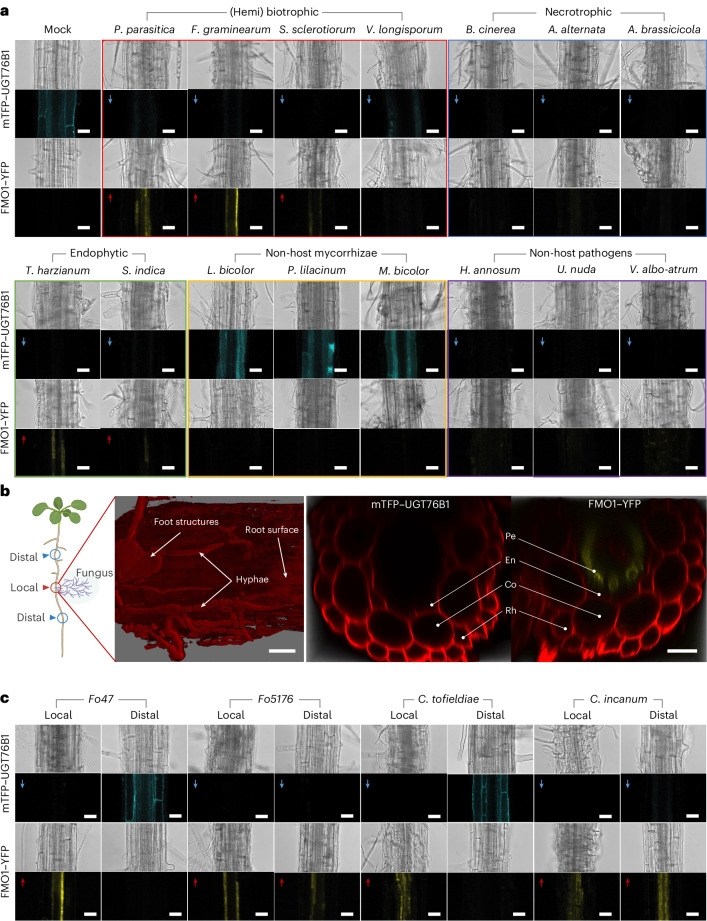
Differential responses of UGT76B1 and FMO1 expression in roots to fungal interactions. **a**, The expression of fluorescently labelled UGT76B1 and FMO1 proteins in response to inoculation with different types of fungi was monitored via confocal microscopy. Two-week-old plants grown on half-strength MS plates were inoculated with fungal plugs next to the root. One or two days after inoculation, roots colonized with fungal mycelium were examined. Blue arrows indicate downregulation of UGT76B1 compared with mock conditions; red arrows indicate upregulation of FMO1. *S. sclerotiorum*, previously classified as a necrotroph, has an early biotrophic phase explaining the observed regulation. Scale bars, 30 µm. **b**, Surface view and cross section of a root infected with *F. culmorum*. Fungal hyphae and root cells are visualized via propidium iodide staining. The expression of UGT76B1 in the cortex and endodermis is absent, while FMO1 is strongly induced in pericycle cells. Pe, pericycle. Scale bars, 20 µm. **c**, UGT76B1 and FMO1 signal in local and distal root areas inoculated with beneficial or pathogenic fungi examined via confocal microscopy; distal regions are taken from uninfected root areas about 1 cm up/downstream of the fungal inoculation. Fo47, *Fusarium oxysporum* 47; Fo5176, *Fusarium oxysporum* 5176. Scale bars, 30 µm. All experiments were repeated twice with similar results.

To understand the biological significance of the constitutive expression of UGT76B1 and FMO1 in roots, we examined their responses to various soil-borne microorganisms. Microorganism-inoculated roots of *UGT76B1*_*pro*_::*mTFP-UGT76B1* and *FMO1*_*pro*_::*FMO1-YFP* plants were examined via confocal microscopy. In mock-treated plants, mTFP–UGT76B1 was present in the cortex and endodermis, whereas FMO1–YFP was barely detectable without enhanced camera sensitivity (Fig. 4a). Upon interaction with endophytic fungi, including three *Trichoderma* species and *Serendipita indica*, mTFP–UGT76B1 signals disappeared following root colonization by hyphae, whereas FMO1–YFP was induced in the pericycle with varying intensities (Fig. 4a,b). Similarly, inoculation with (hemi)biotrophic fungi including three *Fusarium* species, *Phytophthora parasitica* and *Sclerotinia sclerotiorum* led to enhanced FMO1–YFP paralleled by decreased mTFP–UGT76B1 expression. In contrast, necrotrophic pathogens such as *Botrytis cinerea* and two *Alternaria* species caused UGT76B1 suppression without FMO1 induction. This pattern was also observed upon inoculation with three non-host pathogens, including the tree pathogens *Heterobasidion annosum* and *Verticillium albo*-*atrum* and the wheat pathogen *Ustilago nuda*. Additionally, inoculation with non-host mycorrhizae *Laccaria bicolor*, *Purpureocillium lilacinum* and *Meliniomyces bicolor* had no obvious effect (Fig. 4a). To confirm the spatial expression of FMO1, an *F. culmorum*-infected root was analysed showing that FMO1–YFP induction was restricted to the pericycle (Fig. 4b).

Since both endophytes and (hemi)biotrophs lead to similar responses, we selected two pairs of functionally divergent fungi from representative genera and species. Upon inoculation, we examined both the local sites of hyphae-colonized roots and distal, still-uninfected parts of the roots. The *Arabidopsis* beneficial root endophyte *Colletotrichum tofieldiae* (*Ct*) altered UGT76B1 and FMO1 expression only locally, whereas its pathogenic relative, *C. incanum* (*Ci*), led to the loss of mTFP–UGT76B1 and induction of FMO1 even in the distal parts of the root. A similar situation was observed with the beneficial *F. oxysporum* strain *Fo47* and the pathogenic strain *Fo5176* (Fig. 4c).

**Extended Data Fig. 5:**
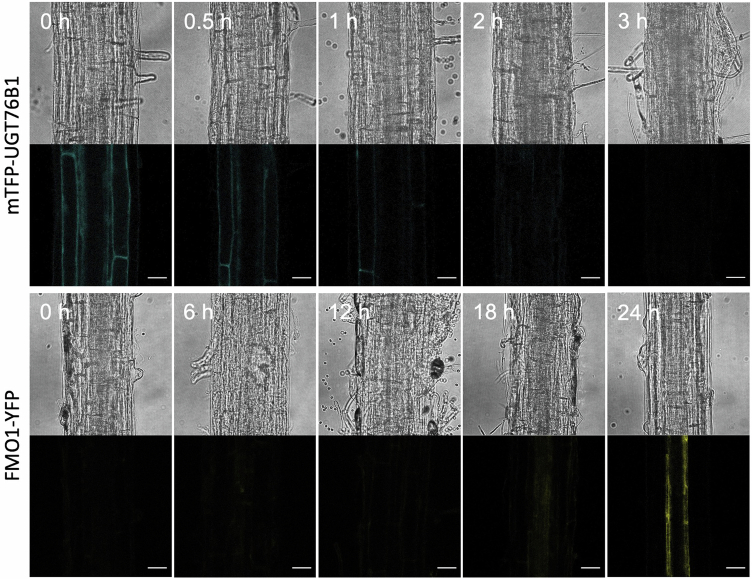
Time course observation of UGT76B1 downregulation and FMO1 induction. Roots of mTFP-UGT76B1 and FMO1-YFP transgenic lines were observed in response to *T. harzianum*. An agar plug with mycelium was placed next to the root, allowing newly emerged hyphae to reach the root immediately. The mTFP-UGT76B1 signal began to fade within 1 h post-inoculation and was completely lost by 3 h. In contrast, FMO1-YFP was induced and became visible after 18 h. Bar, 30 µm. An argon laser was used as the light source. mTFP was excited at 458 nm, with emission collected between 482 and 502 nm (laser intensity: 25%). YFP was excited at 514 nm, with emission collected between 520 and 540 nm (laser intensity: 40%). Imaging was performed using a 40× objective lens. The experiment was independently repeated twice with similar results.

**Extended Data Fig. 6:**
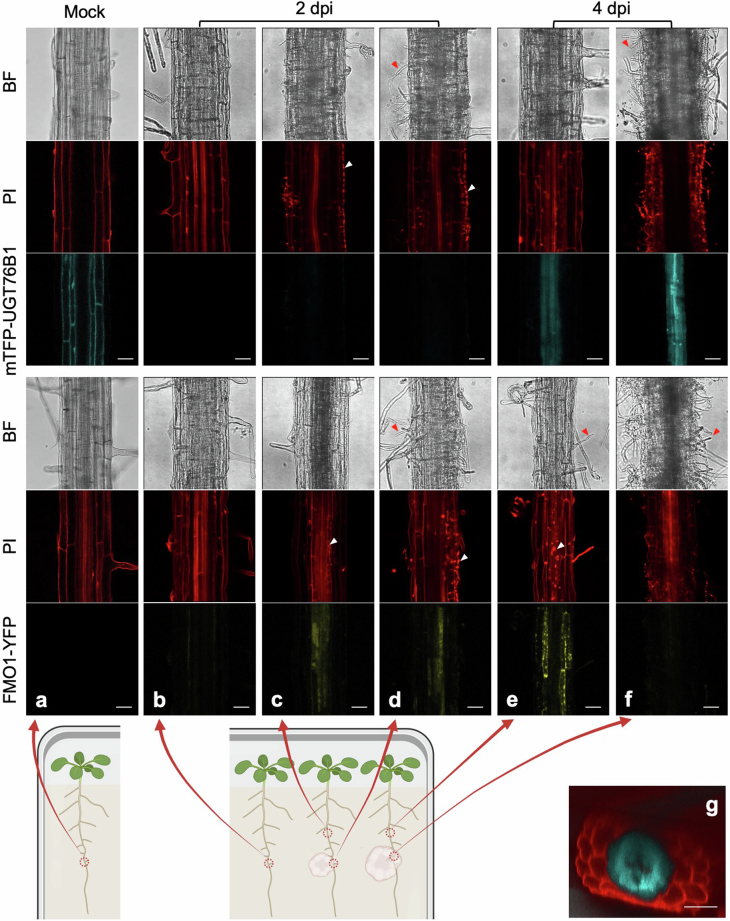
Long-term infection with *C. incanum* (Ci). **a**, Mock-treated plants: mTFP-UGT76B1 is expressed in the cortex and endodermis, while FMO1-YFP is too weak to be detected at the chosen settings. **b**, Plants grown on plates inoculated with *Ci* for 2 days show complete loss of mTFP-UGT76B1 expression, even without direct contact to the fungus. **c-d**, Roots at distal and local sites of inoculation, 2 days post-inoculation (dpi). White arrows indicate fungal nuclei stained by propidium iodide. *Ci* penetrates the root, inducing FMO1 expression in the pericycle while keeping UGT76B1 expression suppressed. **e-f**, Roots at distal and local sites of inoculation, 4 dpi. **g**, mTFP-UGT76B1 expression in the stele. UGT76B1 is induced in the stele, while FMO1 expression in the pericycle disappears in severely infected roots and appears in the cortex and endodermis at regions distal to the inoculation site. BF, bright field; PI, propidium iodide staining. Bar, 30 µm. The experiments were conducted once.

**Extended Data Fig. 7:**
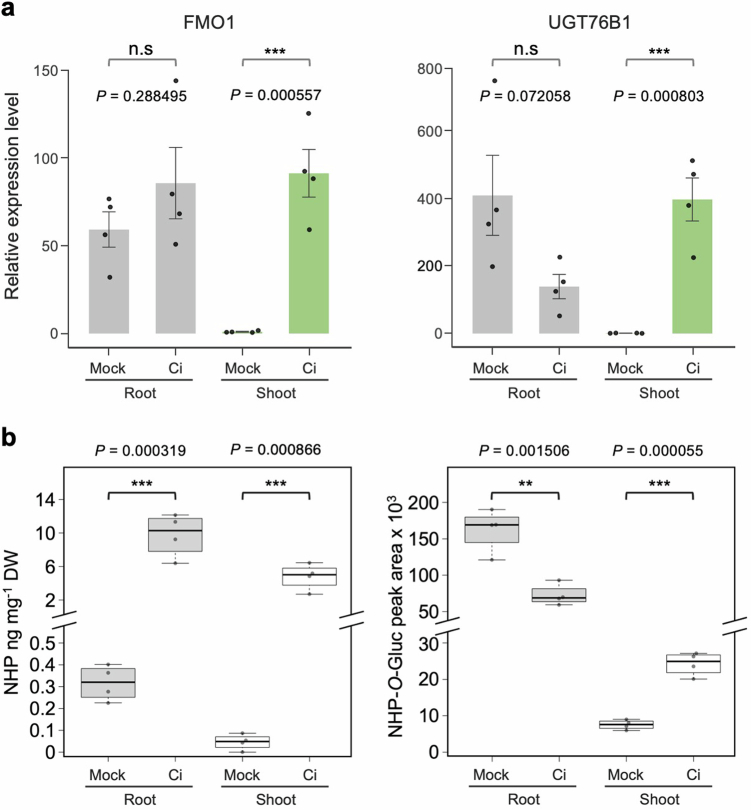
Regulation of *UGT76B1*/*FMO1* expression and NHP metabolites upon *Ci* inoculation. Roots of 3-week-old *Arabidopsis* plants grown on half-strength MS plates were inoculated with *Ci* conidia. Each root was treated with 50 µl of conidia solution containing 10⁶ conidia/ml, evenly distributed on the root surface. Roots and shoots were harvested separately two days post-inoculation. To obtain sufficient root material, 25 plants were pooled per biological replicate. **a**, *UGT76B1* and *FMO1* expression levels were normalized to *S16* and *UBQ5*. Expression in roots only supports a tendency of upregulation of *FMO1* and downregulation of *UGT76B1*, respectively; however, these changes are in line with a local response leaving other parts of the harvested root unaffected. Bars represent means ± standard error, *n* = 4. Root in grey and shoot in green. **b**, NHP and NHP-*O*-Gluc content in roots and shoots with or without *Ci* inoculation. Box in grey represent for root, white for shoot. Boxes represent the interquartile range (Q1–Q3), with the median shown as a bold line. Whiskers extend to 1.5 × IQR, *n* = 4; this analysis was performed once with four independent samples. Significant differences between mock and *Ci*-inoculated tissues were analyzed using the Welch two-sample t-test (*p < 0.05, **p < 0.01, ***p < 0.001; n.s., not significant). The experiments (**a, b**) were conducted once with four independent biological replicates. Source data

To investigate the dynamics of UGT76B1 and FMO1 regulation, root samples were monitored at multiple time points after inoculation with the fast-growing endophyte *Trichoderma harzianum*. mTFP–UGT76B1 rapidly disappeared, becoming undetectable within three hours, while FMO1–YFP induction appeared only after 18 h (Extended Data Fig. 5). To further explore this switch, we examined roots two and four days after *Ci* inoculation. The initial induction of FMO1–YFP in the pericycle disappeared in severely infected roots. mTFP–UGT76B1 expression did not recover in the cortex and endodermis but was instead induced in the stele (Extended Data Fig. 6). This regulation of the proteins in roots was also reflected at the transcript and metabolite levels in roots and shoots. *UGT76B1* transcripts were downregulated in roots after *Ci* inoculation, while both genes were upregulated in shoots. Expression data from several root–microbe/plant interactions corroborate the modulation of *UGT76B1* and *FMO1* (Supplementary Table 1). Consistently, NHP levels were significantly higher in roots and shoots of inoculated plants, whereas NHP-*O*-Gluc accumulated in shoots and decreased in roots (Extended Data Fig. 7a,b).

**Extended Data Fig. 8:**
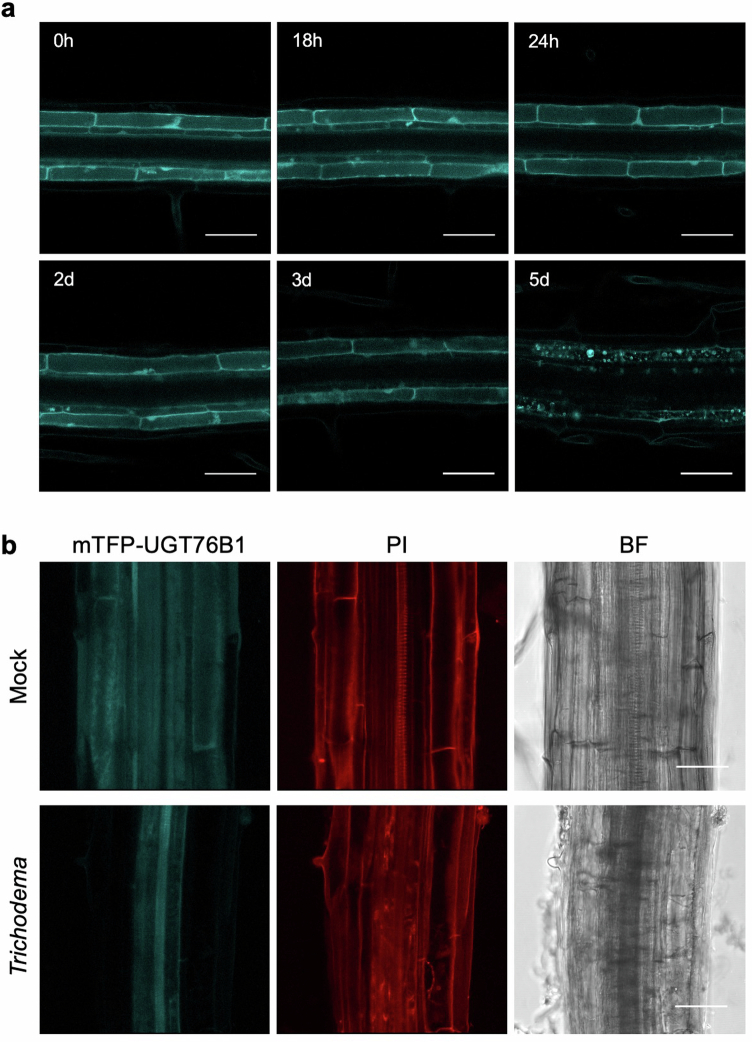
Life span and active degradation of UGT76B1 protein. **a**, Longitudinal sections of a root from a two-week-old plant grown in hydroponic culture, with 100 µM cycloheximide added to the medium to prevent protein synthesis. The mTFP-UGT76B1 signal remained strong in the root even after 3 days of treatment. Five days after the addition of the inhibitor, cell collapse began. Bar: 50 µm. **b**, UGT76B1 is constitutively present in all layers of root cells in the *ugt76b1 35S*_*pro*_*::mTFP-UGT76B1* overexpression line. Upon interaction with *Trichoderma*, the mTFP signal is lost in the outer root cell layers within 1 day, arguing for a rapid degradation of the *per se* stable protein (panel **a**). In contrast, some signal remains in the stele, where *T. harzianum* hyphae may not be able to penetrate. BF, bright field; PI, propidium iodide staining. Bar, 30 µm. Both experiments were repeated twice with similar results.

To determine whether the rapid loss of mTFP–UGT76B1 upon microbial interaction resulted from accelerated degradation or repressed translation of a high-turnover protein, we inhibited protein synthesis using cycloheximide. Under these conditions, mTFP–UGT76B1 remained stable for at least three days. Furthermore, a constitutively expressed mTFP–UGT76B1 disappeared one day after *Trichoderma* inoculation (Extended Data Fig. 8a,b). Thus, the rapid response to microorganisms is probably due to actively initiated degradation. Overall, both FMO1 and UGT76B1 are specifically and frequently oppositely regulated during interactions with different soil microorganisms.

### Dosage effect of NHP on plant growth and defence

Both endophytes and (hemi)biotrophs probably manipulate root NHP levels by modulating FMO1 and UGT76B1 expression. In addition to impacts on defence, endophytes used in this study have been shown to promote plant growth. High endogenous NHP leads to retarded growth^32–34,45^, also by downregulation of growth-related genes^26^, while exogenous NHP feeding has a dosage-dependent effect on shoot defence level^39^.

**Fig. 5:**
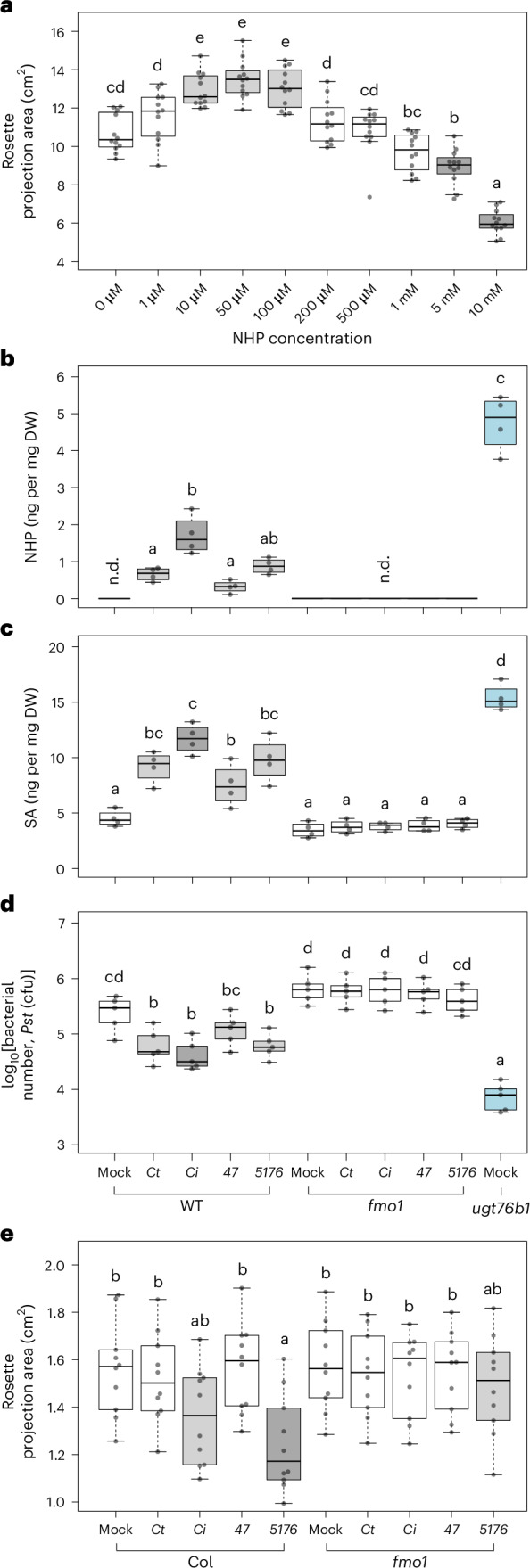
Fungal inoculation effects on growth and immunity mediated by NHP. **a**, Plants were cultivated on soil supplemented with varying concentrations of NHP. The *x* axis represents different concentrations of NHP; 2 ml of NHP solution was supplied around the roots twice a week. Growth was assessed by recording the rosette projection area and fresh weight three weeks post-treatment (Extended Data Fig. 9). The results indicate that low concentrations of NHP stimulate plant growth, whereas high concentrations inhibit it. *n* = 12. Grey boxes indicate groups significantly different from the mock. **b**–**d**, Four-week-old WT and *fmo1* plants were root-inoculated with different fungi; *ugt76b1* (blue) was used as a reference. Shoots were harvested for analysis. WT plants inoculated with different root fungi showed enhanced leaf NHP levels, which were undetectable in mock-treated WT and *fmo1* plants (**b**). *ugt76b1* exhibited high levels of NHP. *47*, *Fusarium oxysporum* 47; *5176*, *Fusarium oxysporum* 5176. Root inoculation triggered leaf SA accumulation in the WT but not in *fmo1* (**c**). Four days after fungal inoculation, leaves of the WT and *fmo1* were challenged with *Pst* DC3000 (**d**). WT plants inoculated with *Ct*, *Ci* and *5176* exhibited stronger resistance than mock-treated WT plants, an effect abolished in *fmo1* plants. *n* = 4. **e**, One-week-old plants subjected to fungal inoculations displayed differential growth responses; the rosette projection area at day 7 post-inoculation is shown. The WT exhibited suppressed growth when inoculated with pathogenic fungi, whereas *fmo1* was unresponsive to fungal inoculation. *n* = 10. The experiments were repeated three times (**a**) or twice (**b**–**e**) with similar results. The boxes represent the IQR (Q1–Q3), with the median shown as a bold line. The whiskers extend to 1.5 × IQR. Significant differences between genotypes and/or treatments were analysed using one-way (**a**) or two-way (**b**–**e**) ANOVA with post hoc Tukey’s test (**a**,**d**,**e**) or the Lincon test (**b**,**c**), as indicated by the letters (*P*_adj_ < 0.05). Source data

**Extended Data Fig. 9:**
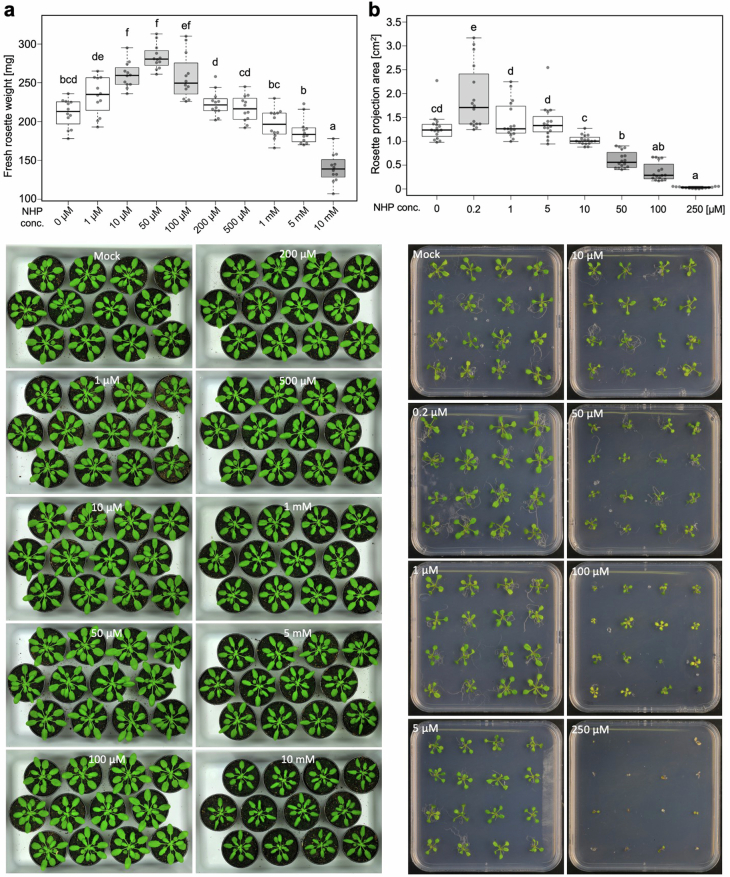
Dosage-dependent effect of NHP. **a**, WT plants grown on soil were watered with 2 ml of NHP at different concentrations twice a week. Fresh weights are measured after four weeks in parallel to the recording of projected leaf area (Fig. 5a). The comparison was performed once. Both analyses resulted in similar recording of plant growth. **b**, Plants were grown on half-strength MS plates supplemented with different concentrations of NHP. The rosette projection area of three-week-old plants was recorded. The results indicate that low concentrations of NHP stimulate plant growth, whereas high concentrations inhibit it, with 250 µM NHP being lethal on plates. Grey boxes indicate groups significantly different from the mock. Boxes represent the interquartile range (Q1–Q3), with the median shown as a bold line. Whiskers extend to 1.5 × IQR, *n* = 16. significant differences were analyzed by one-way ANOVA with post-hoc Lincon test as indicated by letters. Leaf projection areas were measured using ImageJ. Both experiments were independently repeated twice with similar results. Source data

We therefore hypothesized that microorganism-modulated root NHP may have a dosage-dependent effect on plant growth and defence. To test this, we supplied different concentrations of NHP to soil-grown plants. We observed that low concentrations of NHP promoted plant growth, whereas high concentrations suppressed it (Fig. 5a and Extended Data Fig. 9a). A similar trend was observed when plants were grown on NHP-supplemented agar plates (Extended Data Fig. 9b).

**Extended Data Fig. 10:**
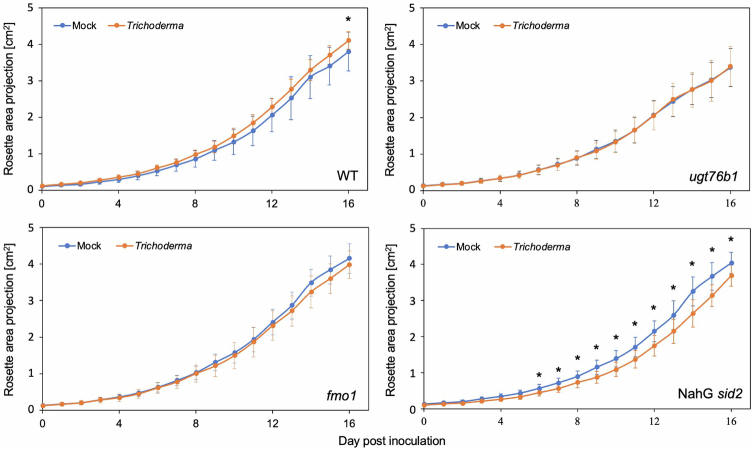
Growth promotion by *T. harzianum* is NHP- and SA-dependent. One-week-old plants were inoculated with 5 ml of a solution containing 10^6^ conidia ml^−1^. Leaf projection area was determined based on imaging regions with active chlorophyll fluorescence, measured daily at 6 a.m. Compared to mock treatment, inoculation with *Trichoderma* significantly enhanced growth of WT plants, an effect not observed for *fmo1* and *ugt76b1* mutants. In SA-depleted plants (NahG *sid2*), the growth response to *Trichoderma* inoculation was lost and even reverted. Alonso-Ramírez et al.^51^ have shown that *sid2* mutants are over-colonized by *T. harzianum*, it colonizes the vasculature and finally kills the plant. SA is important for the plant to keep *Trichoderma* in a beneficial rather than a pathogenic state (Poveda et al., 2023). *ugt76b1* has very high endogenous SA and NHP level (Bauer et al.^33^), which may also prevent *Trichoderma* from accessing the root. *ugt76b1* and *fmo1* both show abolished growth promotion by *Trichoderma*, which supports the notion that NHP is also critical for the interaction. Brotman et al.^23^ showed an induction of *FMO1* transcripts in root upon *Trichoderma*. *fmo1* mutant is also stronger infested by *Trichoderma*. NHP seems as to be as important as SA in the interaction of both species. Thus, UGT76B1’s activity towards NHP and SA may affect the interaction. However, the high SA level is dependent on MHP (FMO1) (Bauer et al.^33^). n = 10, error bars represent standard deviation. Differences between mock and *Trichoderma* inoculation in each time point were analysed by Welch two-sample t test; *p < 0.05. The experiments were independently repeated twice with similar results. Source data

To determine whether some endophyte-induced growth promotion depends on NHP, we inoculated WT, *fmo1*, *ugt76b1* and NahG *sid2* with the plant growth–promoting fungus *T. harzianum* and monitored their growth. After 18 days, WT plants exhibited a significantly larger rosette area than mock-treated controls, while no growth promotion was observed for *fmo1* and *ugt76b1* mutants. NahG *sid2* plants showed suppressed growth upon *T. harzianum* inoculation (Extended Data Fig. 10).

To confirm that NHP mediated the root-microorganism-triggered shoot defence, we inoculated the roots of WT and *fmo1* plants with *Ct*, *Ci*, *Fo47* or *Fo5176*. Rosettes were harvested for metabolic analysis three days post-inoculation. NHP was found in WT shoots inoculated with fungi but undetectable in mock-treated and *fmo1* plants (Fig. 5b). Similarly, endogenous SA levels were elevated only in fungus-inoculated WT plants (Fig. 5c). Plants were also infected by *Pst* four days after fungal inoculation. The WT exhibited enhanced resistance, which was abolished in *fmo1* (Fig. 5d).

To evaluate the early response to root-associated microorganisms, one-week-old seedlings were inoculated with conidia from four different fungi, and their growth was monitored. One week post inoculation, prior to the pathogens entering the necrotrophic phase and causing visible disease symptoms, WT plants inoculated with the pathogen *Fo5176* exhibited a significant reduction in growth. Inoculation with *Ci* also led to a numerically lower rosette area, whereas endophytic fungi *Ct* and *Fo47* did not alter growth. This phenomenon was also abolished in *fmo1* plants (Fig. 5e). In summary, certain root endophytes trigger shoot immunity and promote growth via NHP, whereas (hemi)biotrophs may lead to higher NHP levels, stronger immunity and retarded growth.

## Discussion

**Fig. 6:**
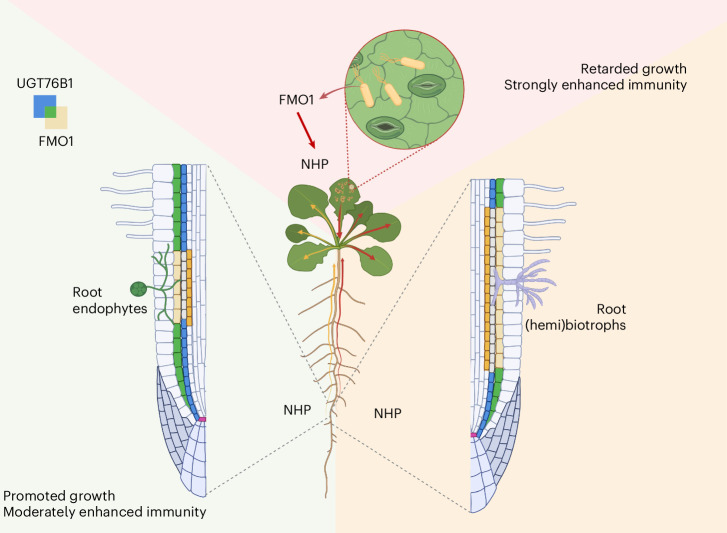
Root-triggered systemic resistance. FMO1 is not expressed in leaves of naïve plants. Upon pathogen attack, FMO1 is induced, and NHP is synthesized de novo. NHP can move systemically to enhance immunity also in distant leaves, a phenomenon known as SAR. In contrast, NHP is continuously synthesized and deactivated in roots due to the simultaneous presence of FMO1 and UGT76B1 establishing a signalling module on hold. Microorganisms differentially affect this ‘inactive’ NHP standby circuit. Endophytic fungi suppress UGT76B1 and promote FMO1 strictly at sites of root–fungus interaction. Biotrophic pathogens elicit a similar response but across a more extended area around the site of interaction. These scenarios lead to different amounts of mobile NHP released from root to shoot; a low level of translocated NHP leads to promoted growth and moderately enhanced immunity, whereas higher levels of NHP provoke retarded growth and more strongly enhanced immunity. Exclusive and overlapping expression of UGT76B1 and FMO1 is shown by the indicated colour code. Figure created with BioRender.com.

Roots are vital for land plants to provide physical support and to acquire nutrients and water. Roots also exchange information with shoot tissue in a reciprocal manner. Among these interactions, induced systemic resistance sensu stricto is a well-studied mechanism on how root-interacting microorganisms establish JA- and ET-dependent and *PR*-gene-independent resistance against pathogens and herbivores in shoots^1,46^. However, other instances, even among the same microbial genus, do not follow these hallmarks, and root-induced shoot resistance depends on SA^14,47^ (Supplementary Table 1). Yet, in both cases, the original trigger emanating from roots remains largely unknown. Here we show that numerous root-interacting fungi exploit components of the leaf-to-leaf SAR, but in a different setting. While SAR is established in leaves by initiating FMO1 expression upon a primary infection to produce the NHP signal, which is later attenuated by the induction of the NHP-conjugating UGT76B1, FMO1 and UGT76B1 exhibit a high basal expression level in naïve *Arabidopsis* roots to synthesize NHP and to concurrently confine its mobility via glucosylation. Upon contact with specific soil microorganisms, this balance is rapidly shifted by the suppression of UGT76B1 and/or upregulation of FMO1. The leaf SAR mechanism of switch-on and keep-in-check is thus altered in case of this root-triggered systemic resistance (RSR) into a standby mode with parallelly active FMO1 and UGT76B1 (Fig. 6). Interestingly, both beneficial and pathogenic microorganisms use this FMO1/UGT76B1 module, albeit with varying intensity. In contrast, non-host mycorrhizae used in this study did not cause effects, probably due to their lack of interaction with *Arabidopsis* roots. We suggest that microbial stimuli at the root are thereby integrated to affect shoot growth and/or defence status via the same principal mechanism. This is supported by the dose-dependent action of NHP not only to activate defence^39^ but also to induce rather than suppress growth at lower NHP levels (Figs. 5 and 6). In naïve plants, the suppression of the shoot defence status is dependent on root-expressed UGT76B1, since *ugt76b1*/WT grafts showed WT-like *PR* gene expression in shoots (Fig. 2a). Thus, the suppressed NHP release from the WT roots does not overcome a defence-activating threshold in leaves despite the absence of UGT76B1.

Beneficial endophytes such as *Fo47* and *Ct* interacting with mature roots usually do not enter the vasculature^22,48^ and lead to localized regulation of FMO1 and UGT76B1 at the root–microbe interface, releasing low amounts of NHP to the shoot to promote growth and moderately enhance resistance. In contrast, some (hemi)biotrophic pathogens such as *Fo5176* and *Ci* can colonize the vasculature^22,48^ (Extended Data Fig. 6) and induce additional distal responses, potentially due to the spread of pathogen-associated molecular patterns or phytotoxins^42^ extending FMO1 and UGT76B1 regulation beyond the site of interaction. This probably leads to a stronger release of NHP, causing reduced growth and a significantly heightened SA-related resistance response in shoots (Figs. 5 and 6 and Extended Data Fig. 9). RSR thus shares key signalling components with SAR and provides a mechanistic explanation of previously identified SA-dependent systemic resistance triggered by root-associated microorganisms, such as *F. oxysporum*^49,50^ or *Trichoderma*^51^.

Despite the antagonistic relationship between JA/ET and SA signalling, induced systemic resistance sensu stricto and RSR elucidated here may also be interwoven, since some root endophytes have been reported to activate both pathways in shoots (for example, *T. virens* and *T. atroviride* or the endophyte *P. indica*)^12,52^. Vice versa, different strains of one species, *P. simiae* (*fluorescens*), may activate JA/ET- or SA-dependent immunity^14,46^ (Supplementary Table 1). These findings may well align with our observations of UGT76B1 and FMO1 regulation—for example, still-active JA signalling in *fmo1* mutants may explain the trend to repress rosette growth and to enhance resistance to *P. syringae* upon interaction with *Fo5176*, since resistance against *Fo5176* depends on both SA and JA pathways^53^ (Fig. 5d,e).

The proper function of this standby FMO1/UGT76B1 module confining the export of NHP from naïve roots depends on efficient glucosylation of the constitutively synthesized NHP. It was thus surprising that unconjugated NHP is detectable in roots, although at a comparatively low level^26,33^ (0.5 versus, for example, 10 ng per mg DW 24 h after *Pseudomonas* infection in leaves; Fig. 1). However, several observations suggest that the spatial distribution of NHP is crucial apart from its overall root level. First, FMO1 expression peaks in the cortex, whereas UGT76B1 is strongly expressed in the cortex and in the endodermis—that is, forming a barrier to NHP’s further release to the vasculature. Second, in line with this interpretation, cell-type-specific loss of UGT76B1 in the endodermis, but not in the cortex, fully mimics the shoot phenotype of the *ugt76b1* knockout. Third, FMO1 is induced in pericycle cells by many hemi(biotrophic) and endophytic fungi adjacent to the phloem rather than in its original expression sites (Fig. 4)—that is, NHP production would occur past the main UGT76B1 barrier.

The diverse approaches to root-microorganism-dependent regulation of shoot immunity and growth based on different hormone signalling pathways^54,55^ may reflect microbial diversity and different environmental contexts. The rhizosphere harbours a greater diversity and density of microorganisms than the phyllosphere^56^. Locally, this challenge primarily requires plants to distinguish between beneficial and harmful microorganisms. Soil moisture favours the activity and proliferation of soil microorganisms, enhancing the potential chances and risks at the root interface^57^. Concurrently, moist soils are frequently coupled to higher air humidity and thereby enhanced microbial impact on the shoot organs^58^. Some pathogens may also use the root entry route to colonize aerial parts^59^. A rapid mechanism of root–shoot communication as provided by the root-based standby FMO1/UGT76B1 module of RSR may thus confer an adaptive advantage in a systemic context. Moreover, the very same module in roots can fuel dose-dependent NHP signalling to coordinate the growth and defence status of shoots and is adaptive to different lifestyles, benefits and threats of the interacting microorganisms (Fig. 6).

## Methods

### Plant materials and growth conditions

This study used *Arabidopsis thaliana* accession Columbia (WT) along with mutant and transgenic lines: *ugt76b1-1* (SAIL_1171A11)^36^, *fmo1-1* (SALK_026163)^60^, the SA-depleted double mutant NahG *sid2* (ref. ^36^), *sid2* *ugt76b1*, NahG *sid2* *ugt76b1*, *fmo1* *ugt76b1* (ref. ^61^), *UGT76B1*_*pro*_::*GFP-GUS*^36^, *FMO1*_*pro*_::*GUS*^41^ and the *fmo1-1* complementing *FMO1*_*pro*_::*FMO1-YFP*^42^. Plants were cultivated in a controlled growth chamber under a 10 h light/14 h dark cycle at 22/18 °C, 60/70% relative humidity and 120 µmol m^−2^ s^−1^ light intensity (type 840 fluorescent lamps; Osram). They were grown on a mixture of peat-moss-based substrate (Floragard Multiplication substrate) and quartz sand (12:1). A proportion of 6:1 was used for plant growth and monitoring in the phenotyping facility (Photon Systems Instruments). For fungal inoculation assays, seeds were germinated on half-strength MS medium (Duchefa) supplemented with 0.5% sucrose, stratified at 4 °C for two days and subsequently grown under the same conditions described above.

### *PR* gene expression analysis

RNA isolation, reverse transcription and quantitative PCR were performed according to Bauer et al.^33^ to assess the transcript levels of *PR1*, *PR2* and *PR5* (Supplementary Table 3).

### Fluorescent-protein-labelled *ugt76b1* complementation line

The transgenic line *UGT76B1*_*pro*_::*mTFP-UGT76B1* was generated to complement the *ugt76b1-1* mutant by expressing an amino-terminal mTFP fusion of *UGT76B1* under the control of its native promoter. A Gibson assembly reaction (New England Biolabs) was used to fuse three fragments: (1) a 1,754-bp *UGT76B1* promoter region, (2) the mTFP coding sequence without a stop codon and (3) a *UGT76B1* gene segment including the ATG start codon and 505 bp of the 3′ untranslated region (Supplementary Table 3). This construct was recombined via pDONR221 (Invitrogen; screen with 50 mg l^−1^ kanamycin) into pAlligator2Δ35S (screen with 100 mg l^−1^ spectinomycin), a modified version of pAlligator2 with the CaMV 35S promoter removed^62^. The deletion was achieved via restriction enzyme digestion with EcoRI and HindIII, followed by blunt-ending using T4 DNA ligase and religation. The final vector was used for *Agrobacterium*-mediated transformation of *ugt76b1-1* via the floral dip method^63^. Segregation analysis identified two independent homozygous transgenic lines with single insertions.

### TSKO

UGT76B1 cortex- and endodermis-specific knockout lines are based on the *UGT76B1*_*pro*_::*mTFP-UGT76B1* complementation line. Plasmids for tissue-specific genome editing were generated via PCR amplification and GoldenGate cloning employing BsaI and BpiI (ThermoFisher)^64^. Regulatory sequences of At2g36100 (*CASP1*) and At1g62500 (*Co2*) were amplified via PCR (Supplementary Table 3) and cloned into pAGM1251 (ref. ^65^) via BpiI restriction/ligation to yield pCK256 and pCK257. Subsequently, promoter elements were assembled with (NLS)mCherry-P2A (pCK237), zCas9i (pCK70) and rbcs-E9 (terminator, pJOG416) modules in pICH47742 to yield pCK259 and pCK260. These modules were further assembled in the Level 2 acceptor pJOG292 (ref. ^66^) together with a BsaI-excisable *ccdB* cassette, the FAST seed fluorescence marker^67^ and either spraying 1:800 diluted commercial Basta for soil-grown plants (*CASP1*) or screening in half-strength MS medium containing 30 mg l^−1^ hygromycin (*CO2*) resistance cassette to yield pDGE1075 and pDGE1076, respectively. To generate the final plant transformation vectors, sgRNA-coding oligonucleotides were cloned into the sgRNA shuttle vectors pDGE332 and pDGE334 (Supplementary Table 3), and the assembled sgRNA transcriptional units were mobilized into pDGE1075 or pDGE1076 to yield pDGE1075-B1en and pDGE1076-B1co. These binary vectors were transformed into *Agrobacterium tumefaciens* GV3101 pMP90 for plant transformation.

### Histochemical analyses

For histochemical analyses of promoter–GUS reporter lines, plant tissues were stained^68^ for 30 min (*UGT76B1*_*pro*_ plants) or 12 h (*FMO1*_*pro*_ plants). Chlorophyll was removed by destaining with 70% ethanol. Images were captured using a stereomicroscope (Zeiss Stemi 2000-C) at ×20 magnification. Protein expression of *UGT76B1* and *FMO1* in roots was visualized using the *UGT76B1*_*pro*_::*mTFP-UGT76B1* and *FMO1*_*pro*_::*FMO1-YFP* lines, respectively, with a confocal laser scanning microscope (SP8, Leica). For cell wall staining, two-week-old seedlings grown on vertical agar plates were treated with 50 μg ml^−1^ propidium iodide for 30 min and then rinsed twice with double-distilled water before imaging. An argon laser was used as the light source. mTFP was excited at 458 nm, with emission collected between 482 and 502 nm (laser intensity, 25%; 100% gain; pinhole set to 1). YFP was excited at 514 nm, with emission collected between 520 and 540 nm (laser intensity, 40%; 100% gain; pinhole set to 2.5). When co-detected with both fluorescent proteins, PI staining shared their excitation wavelengths and was detected between 626 and 646 nm. All confocal images were acquired with a ×40 water immersion objective lens, with an area size of 320 µm × 320 µm and a resolution of 1,024 × 1,024. In Fig. 4, the images were cropped to fit the layout; all other figures retain their original size.

### Micrografting assay

The grafting protocol was adapted from a previous description^69^. Seeds were sterilized and sown on half-strength MS medium without vitamins (Duchefa; 1% sucrose; 1% bacteriological agar, Roth). After two days of stratification, the plates were moved to a growth incubator (MLR 351H, Sanyo) and incubated for three days under constant light (50 μmol m^−2^ s^−1^) at 22 °C; then the light intensity was reduced to 10 μmol m^−2^ s^−1^ for two additional days to promote hypocotyl elongation. Seedlings were cut straight through the middle of the hypocotyls using a fresh razor blade, and rootstocks and scions were combined in the desired combinations on half-strength MS medium with 0.5% sucrose (see Supplementary Fig. 2 for a detailed operation illustration). The grafted seedlings were then grown vertically under constant light (10 μmol m^−2^ s^−1^) at 27 °C for one week, followed by one week at 50 μmol m^−2^ s^−1^ light under short-day conditions (10 h light at 22 °C; 14 h dark at 17 °C). Afterward, the plants were transferred to 120 × 120 mm square Petri dishes (Greiner Bio-One) containing 50 ml of half-strength MS medium without sucrose. Two weeks later, the plants were examined to exclude any fusions that had developed adventitious roots. Whole rosettes were harvested for gene expression analysis. For disease assays, the plants were moved to a slurry soil and grown covered with a lid for two days to maintain high humidity. They were then cultivated under regular conditions for another two weeks before the assay.

### NHP feeding assay

For soil-grown plants, 2 ml of NHP solutions at concentrations of 1 µM, 10 µM, 50 µM, 100 µM, 200 µM, 500 µM, 1 mM, 5 mM and 10 mM were applied twice a week with a pipette to the soil around the roots of plants maintained under short-day conditions. Ten plants were used for each concentration. For plants grown on Petri dishes, seeds were sown on half-strength MS medium supplemented with NHP at concentrations of 0.2 µM, 1 µM, 5 µM, 10 µM, 50 µM, 100 µM and 250 µM (MedChemExpress). After two days of stratification, the plates were transferred to short-day conditions and positioned horizontally, with 16 plants for each concentration. Four-week-old soil-grown plants and 18-day-old plate-grown plants were imaged using an RGB camera, and the rosette area was measured using ImageJ for Mac (version 1.53K) software.

### Microbial culture conditions and inoculation assay

The fungal and oomycete strains used in this study are listed in Supplementary Table 4. Strains were cultured at 22 °C on VJS agar medium^70^ for *S. indica*, *V. longisporum* and *P. parasitica* or on PDA medium (Sigma-Aldrich) for all other strains. For conidia harvesting, corresponding strains were grown in VJS liquid medium or PD broth (Sigma-Aldrich) at 24 °C with shaking at 180 rpm. After four days of cultivation, the liquid cultures were filtered through cheesecloth to remove mycelium. The conidia suspension was then centrifuged at 2,000 *g* for 10 min at 4 °C, and the resulting pellet was washed twice with 10 mM MES buffer (pH 5.8) and resuspended in 0.05% Tween 20 solution. Conidia were counted using a haemocytometer, and the suspension was adjusted to a final concentration of 10^6^ conidia per ml. For plant inoculation, 5 ml of the prepared conidia suspension was applied to the soil around the roots of 12-day-old plants (for growth measurement) and four-week-old plants (for *Pst* disease assays), taking care to avoid direct contact between the conidia and the rosette.

### Bacterial inoculation and infection assays

Fully developed leaves from four- to five-week-old plants were gently infiltrated with either 10 mM MgCl_2_ (as a control) or suspensions of *Pst* DC3000 in 10 mM MgCl_2_. For basal resistance assays, plants inoculated with *Pst* (OD_600_ = 0.0001) were kept under standard growth conditions for three days. Leaf discs from three independent plants (three discs per plant, pooled to form one biological sample) were collected 2 and 72 h post-inoculation and immersed in 500 µl of 10 mM MgCl₂ containing 0.01% Silwet L77 (Momentive; via Obermeier). Bacterial growth was quantified as described previously^71^. Each treatment was replicated five times; the entire experiment was conducted independently twice. To assess root-induced resistance, four-week-old plants were inoculated with 5 ml of conidia suspension as described above, and three days later, the same leaf-based disease assay was performed.

### LC–MS analyses

SA, NHP and NHP-*O*-Gluc were quantified using LC–MS following extraction from freeze-dried plant material^33^. 5 µl of each extract was injected twice as technical replicates for the LC–MS analysis. NHP and NHP-*O*-Gluc were detected using positive ionization mode, while SA was measured in negative ionization mode. Authentic standards for SA (Sigma-Aldrich) and NHP (MedChemExpress) were used for identification and quantification.

### Statistics

All experiments were repeated at least twice except for the data in Fig. 3c and Extended Data Fig. 9, which were added during revision. In all cases, a single experimental series was based on at least four independent biological samples. Statistical analyses were conducted using R version 4.4.1 for Mac (https://www.r-project.org/). For analysis, we employed the WRS2 package, which includes Wilcox’s robust statistical methods. Robust one-way and two-way ANOVA were performed for multiple group comparisons using the t1way and t2way functions, respectively. Prior to selecting post hoc tests, we applied the Shapiro–Wilk and Levene’s tests to assess the normality and homogeneity of variances, which determined the use of either the Lincon test or Tukey’s honestly significant difference for post hoc comparisons. Welch’s two-sample *t*-tests were used for pairwise comparisons between two groups. The types of statistics are indicated in the figure legends.

### Reporting summary

Further information on research design is available in the Nature Portfolio Reporting Summary linked to this article.

## Supplementary information

Supplementary Information Supplementary Figs. 1 and 2 and Tables 1–4.

Reporting Summary

## Source data

Source Data Figs. 1–3 and 5 and Extended Data Figs. 3, 4, 7, 9 and 10 Original data and statistical analyses for Figs. 1b,c, 2a–d, 3b,c and 5a–e and Extended Data Figs. 3, 4a, 7a,b, 9a,b and 10.

## Data Availability

The original LC–MS and confocal microscopic data are available via OSF at 10.17605/OSF.IO/HKX75 (ref. ^72^) and 10.17605/OSF.IO/EV796 (ref. ^73^). Correspondence and requests for materials should be addressed to A.R.S. Source data are provided with this paper.
