# Supplementary material for: A root-based N-hydroxypipecolic acid standby circuit to direct immunity and growth of Arabidopsis shoots
Source: Nat Plants. 2025 Jul 22;11(8):1658–69. doi: 10.1038/s41477-025-02053-2 (PMC12364709; doi:10.1038/s41477-025-02053-2)
Supplement: Supplementary file 1 — Supplementary Figs. 1 and 2 and Tables 1–4. [file 41477_2025_2053_MOESM1_ESM.pdf]

nature plants

Supplementary information

https://doi.org/10.1038/s41477-025-02053-2

A root-based *N*-hydroxypipecolic acid standby circuit to direct immunity and growth of *Arabidopsis* shoots

In the format provided by the authors and unedited

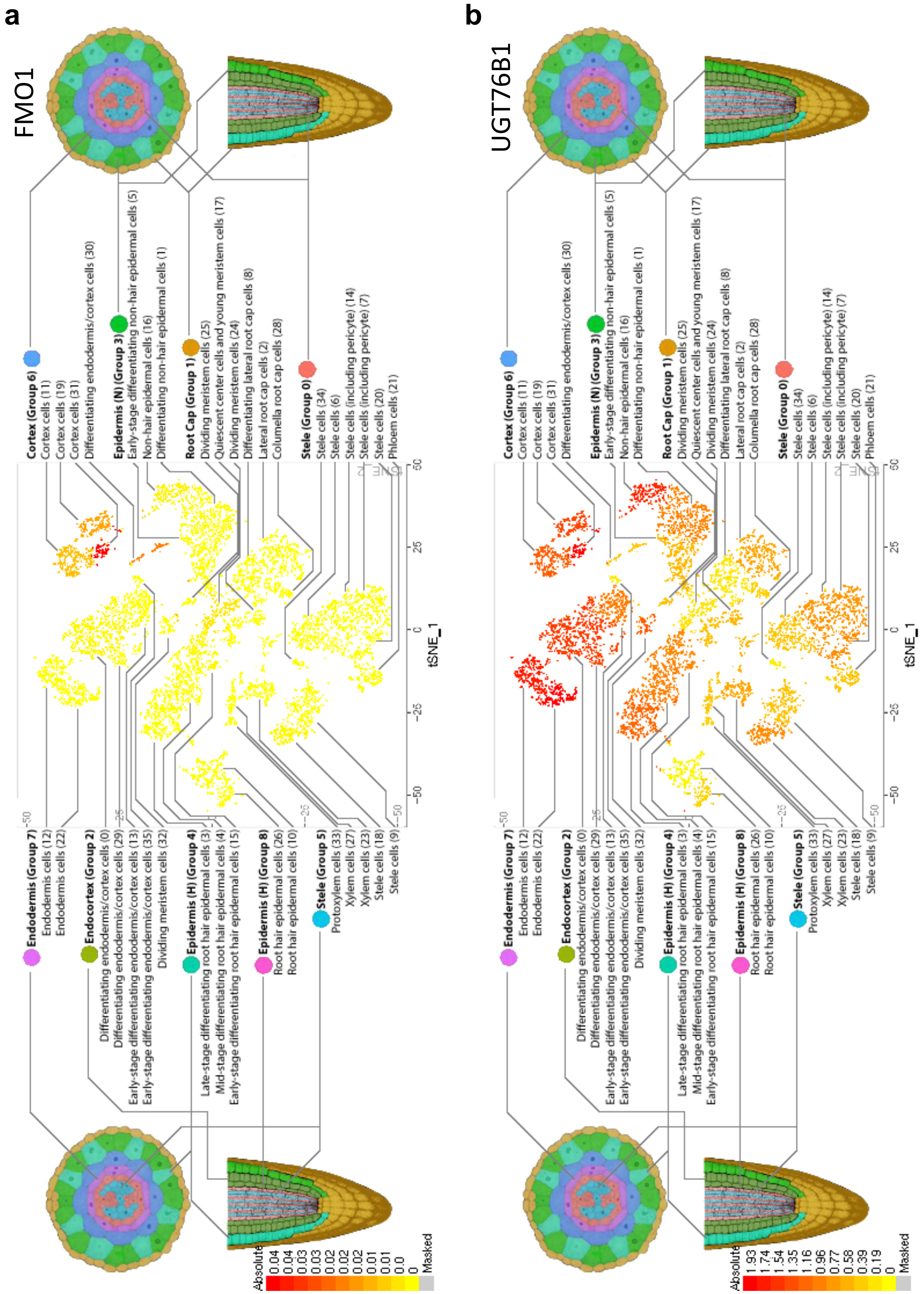

Supplementary Fig. 1 | Root expression profile of *FMO1* and *UGT76B1* by single cell sequencing. **a**, Expression pattern of *FMO1* in the root, showing weak expression in the cortex and differentiating endodermis/cortex cells. **b**, Expression pattern of *UGT76B1* in the root, with strong expression in the cortex and endodermis, and weaker expression in the rhizodermis and stele. The color code represents absolute transcript levels. Images obtained from BAR, ePlant (https://bar.utoronto.ca/eplant/).

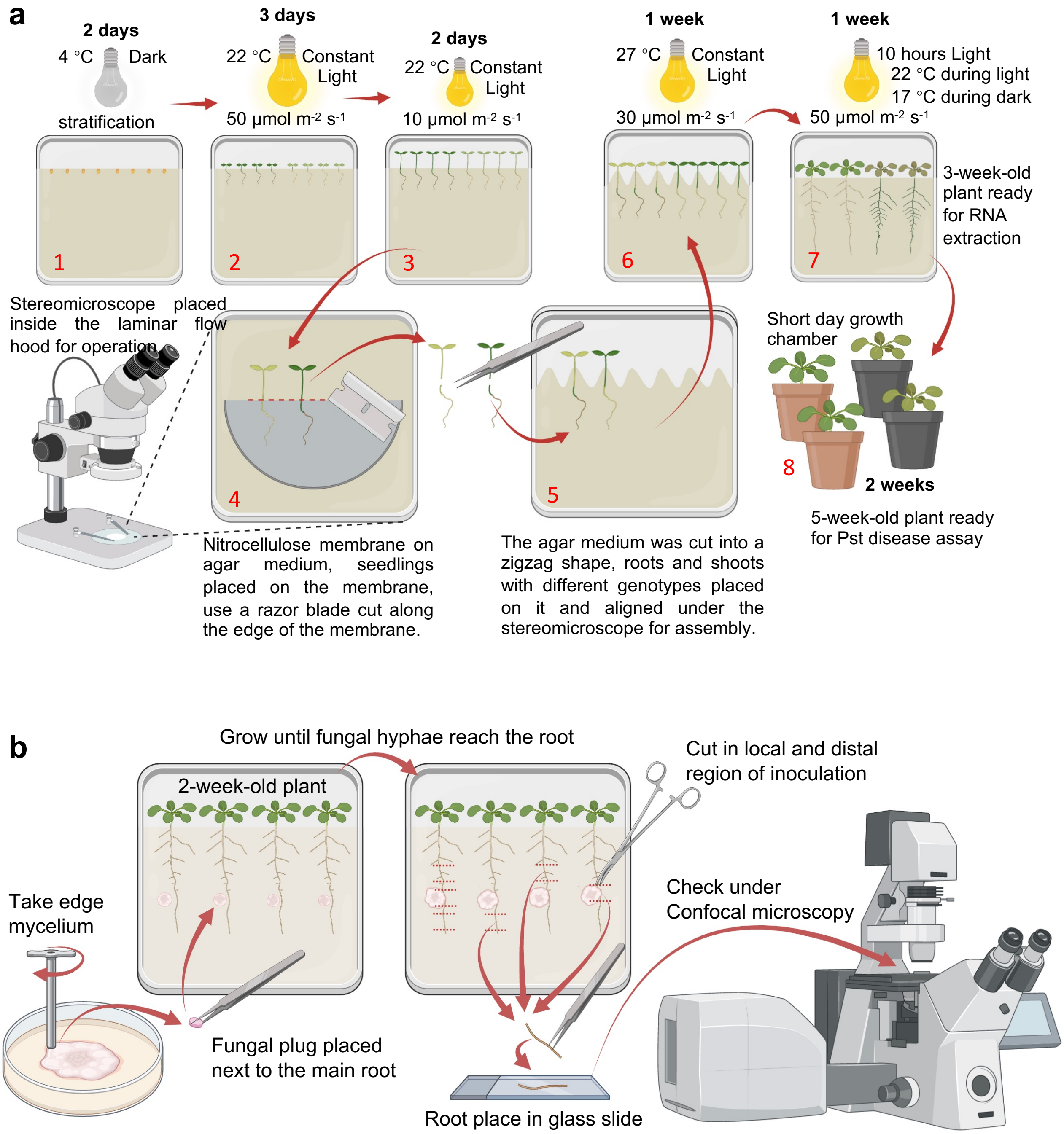

Supplementary Fig. 2 | Illustration of micrografting and root microbe inoculation. a, Micrografting, details see methods. **b**, Root microbe inoculation, details see methods. Created in BioRender. Xu, P. (2025) https://BioRender.com/dxme4sd.

SupplementaryTable 1 | Summary of plant growth promoting fungi and rhizobacterias induced systemic resistance and their hormone dependency.

Species	Strain	Phenotypes of inoculation	Hormone dependency	Marker gene expression in shoot	Reference
Pseudomonas simiae	WCS417r	Enhanced resistance against *Pst*	JA and ET		Pieterse *et al.,* 1998
			SA	Enhanced SA and JA marker genes	Sommer *et al.,* 2024*
Pseudomonas fluorescens	SS101	Enhanced resistance against *Pst*, *P. syringea pv. maculicola*, *P. alisalensis and viridiflava*. However, more susceptible to insect pest *Spodoptera exigua*	SA	Enhance SA marker genes	E. van de Mortel *et al*., 2012
Paenibacillus alvei	K165	Enhanced resistance against *Verticillium dahliae*	SA	Enhance SA marker *PR1/2/5* expression	E. Tjamos *et al.,* 2005
Fusarium equiseti	GF19-1	Enhanced resistance against *Pst*	SA	Enhance SA marker *PR1/2/5* expression	Kojima *et al.,* 2013
Trichoderma asperellum	SKT-1	Enhanced resistance against *Pst*	SA	Both SA and JA marker genes	Yoshioka *et al.,* 2011
Trichoderma hamatum	T382	Enhanced resistance against B. cinerea	SA	Enhanced SA biosynthesis and *PR* genes	Mathys *et al*., 2012
Trichoderma harzianum<br>*Pseudomonas fluorescens*	Tr6<br>Ps14	Combined inoculation of both endophytes promote the resistance *B. cinerea* and *F. oxysporum*	SA		Alizadeh *et al,*. 2013
Trichoderma virens<br>*Trichoderma atroviride*	Gv.29-8<br>IMI 206040	Enhanced resistance against *B. cinerea*		Low dosage of conidia induce JA marker LOX1 while high dosage induces SA marker PR1	Contreras-Cornejo *et al.,* 2011
Trichoderma harzianum<br>*Trichoderma parareesei*	CECT 242<br>CECT 20106	Enhanced resistance against *B. cinerea*		Induced JA marker *LOX1* and suppresed SA marker *PR1*	Poveda, 2021
Trichoderma harzianum<br>*Bacillus amyloliquefaciens*	A<br>I3			Induced JA marker *LOX1* and SA marker *PR1* in both root and shoot	Ilham *et al.,* 2019

"*Hormone dependency*" indicates whether resistance was assessed using mutants deficient in SA, JA, or ET biosynthesis pathways.
"*Marker gene expression*" refers to the expression of SA or JA marker genes in the shoots of plants inoculated with the respective fungi in the root system.
* In this study, Sommer et al. demonstrate that *P. simiae* moves to the shoot after root inoculation, thus they classify it as "locally induced resistance."

SupplementaryTable 1 | Part 2: Transcriptional regulation of SA- and NHP-relatedtranscripts compiled from literature.

For each pairwise comparison, log2 expression fold-changes (logFC) and associated FDR-corrected p-values are indicated, data are only show up if FDR p-value < 0.05.
Genes with a log2FC ≥ 0 are higlighted in blue and genes with a log2FC < 0 are highlighted in Orange. Tissues and microbes are highlighted in Red.

van de Mortel et al., 2012 doi.org/10.1104/pp.112.207324

Differentially expressed genes in *Arabidopsis* roots and leaves treated with *P. fluorescens* SS101 in root

Function	AGI code[a]	gene name	SS101/Control root 10d[b]		SS101/Control root 14d[b]		SS101/Control root 18d[b]		SS101/Control leaves 10d[b]		SS101/Control leaves 14d[b]		SS101/Control leaves 18d[b]	
			FC[c]	FDR p-value	FC[c]	FDR p-value	FC[c]	FDR p-value	FC[c]	FDR p-value	FC[c]	FDR p-value	FC[c]	FDR p-value
P glucosyltran	At3g11340	UGT76B1							17.77	0.01120209	4.25	0.00135342		
NHP biosynthesis genes	At2g13810	ALD1												
	At5g52810	SARD4							2.08	0.03044775	2.15	0.01716665		
	At1g19250	FMO1	2.76	0.01021233			2.27	0.01159239						
SA biosynthesis genes	At1g74710	SID2							2.58	0.00909634				
	At4g39030	EDS5							5.18	0.01234855	3.73	0.00247078		
	At5g13320	PBS3	2.04	0.04305615			2.17	0.03234452	3.24	0.04304148	2.17	0.01039199		
	At5g67160	EPS1												
	At1g18870	ICS2							2.58	0.00909634				
SA-related defense genes	At4g33730	PR1	3.71	0.02287898			4.42	0.01226248	186.36	0.00050353	61.91	0.00310057		
	At3g57260	PR2									2.42	0.04827667		
	At1g75040	PR5							2.31	0.01573214				

[a]AGI gene code (At….). [b]Ratio of significant (FDR<0.05) differential (≥ 2) expressed genes between *Pf* .SS101 treated plants and control (mock treated) plants grown for 10, 14 or 18 days. [C] Fold change between Pf.SS101 treated and mock treated plants.

Weston et al., 2012 doi.org/10.1094/MPMI-09-11-0253

Differentially expressed genes in Arabidopsis roots and leaves after root inoculaion of *P. fluorescens* strain GM30 and Pf5

Function	AGI code	gene name	GM30/Control 3d root		GM30/Control 3d shoot		Pf5/Control 3d root		Pf5/Control 3d shoot	
			FC	FDR p-value	FC	FDR p-value	FC	FDR p-value	FC	FDR p-value
P glucosyltran	At3g11340	UGT76B1								
NHP biosynthesis genes	At2g13810	ALD1								
	At5g52810	SARD4								
	AT1G19250	FMO1	1.03	0.0003017						
SA biosynthesis genes	At1g74710	SID2	0.88	0.00105516	0.42	0.031777544			1.02	0.00205802
	At4g39030	EDS5								
	AT5G13320	PBS3	1.64	5.6815E-06						
	At5g67160	EPS1								
	At1g18870	ICS2	0.68	0.00637873	1.17	0.000163352				
SA-related defense genes	AT4G33730	PR1			1.08	0.044135785				
	At3g57260	PR2								
	At1g75040	PR5								

SupplementaryTable 1 | Part 2: Transcriptional regulation of SA- and NHP-relatedtranscripts compiled from literature.

For each pairwise comparison, log2 expression fold-changes (logFC) and associated FDR-corrected p-values are indicated, data are only show up if FDR p-value < 0.05.
Genes with a log2FC ≥ 0 are higlighted in blue and genes with a log2FC < 0 are highlighted in Orange. Tissues and microbes are highlighted in Red.

Hacquard et al., 2016 10.1038/ncomms11362

A. thaliana differential gene expression during *C. tofieldiae* colonization under phosphate sufficient and deficient conditions

Differentially expressed genes Ct-colonized vs Mock-treated roots)

			**Plus Phosphate**								**Minus Phosphate**							
**Function**	**GeneID**	**Symbol**	**6d LogFC**	**FDR p-value**	**10d LogFC**	**FDR p-value**	**16d LogFC**	**FDR p-value**	**24d LogFC**	**FDR p-value**	**6d LogFC**	**FDR p-value**	**10d LogFC**	**FDR p-value**	**16d LogFC**	**FDR p-value**	**24d LogFC**	**FDR p-value**
P glucosyltran	AT3G11340	UGT76B1	-0.68	0.00001500	-0.65	0.00006269					-0.60	0.00022573	-0.92	0.00000025	-0.54	0.00076028		
NHP biosynthesis genes	AT2G13810	ALD1					-1.00	0.00286794	0.77	0.00673364							1.27	0.00831654
	AT5G52810	SARD4	-0.72	0.00002789			-0.52	0.00115834	0.50	0.00127690							0.75	0.00000687
	AT1G19250	FMO1	3.13	0.00862634			-0.91	0.00504749	1.17	0.00003027	2.21	0.04287018					0.61	0.04074242
SA biosynthesis genes	AT1G74710	SID2							0.56	0.00145686								
	AT4G39030	EDS5	-0.82	0.00001327	-0.42	0.00816216											-0.41	0.00313052
	AT5G13320	PBS3	3.56	0.03179267					1.51	0.00010206	3.47	0.03107844						
	AT5G67160	EPS1	-0.79	0.00000269			-0.44	0.00146107			-0.47	0.00477165	0.44	0.00289256			0.49	0.00065226
	AT1G18870	ICS2	-2.35	0.00014312	-0.92	0.00354630			-1.17	0.00002517			-1.37	0.00001305	-0.95	0.00049187	-2.33	0.00000000
SA-related defense genes	AT4G33730	PR1											0.70	0.04069157	1.42	0.00005476		
	AT3G57260	PR2					2.20	0.73611912	6.97	0.01133252							3.82	0.02950751
	AT1G75040	PR5							6.64	0.00753035							4.05	0.02708826

Pérez-Alonso *et al* ., 2022 doi.org/10.1111/pce.14420

Differentially expressed genes in *Arabidopsis* roots inoculaion of *P. indica*

			**S. indica/Control 2dpi root**		**S. indica/Control 10dpi root**	
**Function**	**AGI code**	**gene name**	**FC**	**FDR p-value**	**FC**	**FDR p-value**
P glucosyltran	At3g11340	UGT76B1				
NHP biosynthesis genes	At2g13810	ALD1			1.66	2.18113E-12
	At5g52810	SARD4				
	AT1G19250	FMO1			2.88	7.74891E-26
SA biosynthesis genes	At1g74710	SID2				
	At4g39030	EDS5				
	AT5G13320	PBS3			3.94	6.88033E-17
	At5g67160	EPS1				
	At1g18870	ICS2				
SA-related defense genes	AT4G33730	PR1				
	At3g57260	PR2				
	At1g75040	PR5				

**SupplementaryTable 1 | Part 2: Transcriptional regulation of SA- and NHP-relatedtranscripts compiled from literature.**
For each pairwise comparison, log2 expression fold-changes (logFC) and associated FDR-corrected p-values are indicated, data are only show up if FDR p-value < 0.05. Genes with a log2FC ≥ 0 are higlighted in blue and genes with a log2FC < 0 are highlighted in Orange. Tissues and microbes are highlighted in Red.

Martínez-Soto et al., 2023 doi.org/10.1094/MPMI-08-22-0166-SC

		FPKM after root inoculation of *Fusarium*			
		**12 hpi**	**24 hpi**	**48 hpi**	**96 hpi**
**UGT76B1**	Fo47	138.66	121.52	144.64	139.83
	Fo5176	171.73	96.00	173.20	173.01
**FMO1**	Fo47	4.38	17.28	35.34	88.52
	Fo5176	4.33	8.23	56.76	82.79

FPKM values were averaged across three biological replicates

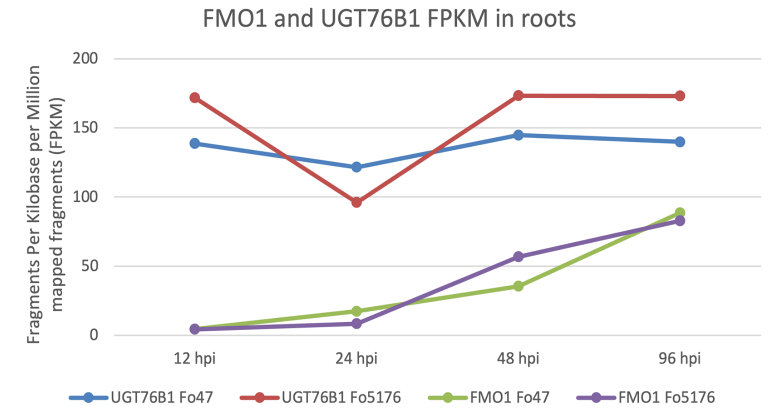

Brotman et al., 2013 doi.org/10.1371/journal.ppat.1003221

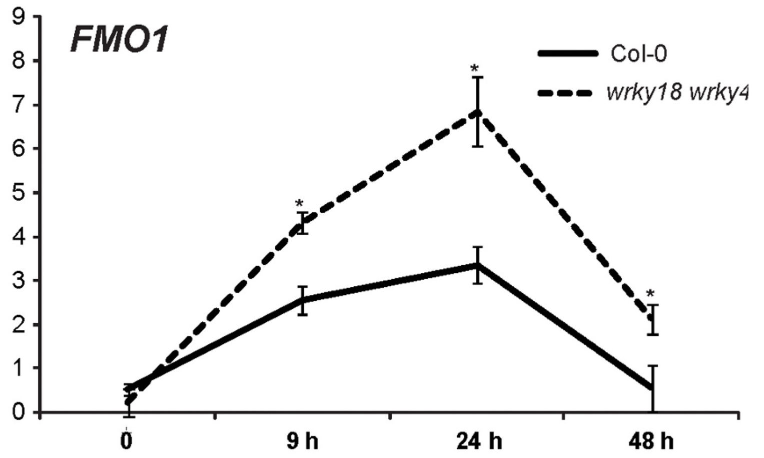

Expression of Arabidopsis genes after root colonization by T. asperelloides. The expression of several genes as determined by qPCR in WT (solid lines) and wrky18/wrky40 (dashed lines) plants at 9, 24 and 48 h hpi. Gene expression level was calculated with respect to control at each time point. Each time point represents the fold expression average of three independent biological repetitions, and is relative to control collected in each of the specified time points. * significant different (P,0.05; t test).

Supplementary Table 2 | Coexpression between *UGT76B1* and NHP-, SA-biosynthetic genes.

Gene name	AGI Code	Pathway	Gene name	AGI Code	coex z
UGT76B1	AT3G11340	NHP biosynthesis	*FMO1*	AT1G19250	4.1
			ALD1	AT2G13810	4
			SARD4	AT5G52810	3.5
		SA biosynthesis	*SID2*	AT1G74710	2.8
			EDS5	AT4G39030	2.5
			PBS3	AT5G13320	1.4
			EPS1	AT5G67160	0.6
			ICS2	AT1G18870	-0.7

ALD1, AGD2-LIKE DEFENSE RESPONSE PROTEIN1; **SARD4**, SAR-DEFICIENT4; **SID2**, SA INDUCTION-DEFICIENT2; **EDS5**, ENHANCED DISEASE SUSCEPTIBILITY5; **PBS3**, avrPphB SUSCEPTIBLE3; **EPS1**, ENHANCED PSEUDOMONAS SUSCEPTIBILITY; **ICS2**, ISOCHORISMATE SYNTHASE2. Data obtained from ATTED-II: ath-m.c9-0 ath-r.c5-0 (https://atted.jp/top_draw/#CoexViewer).

Supplementary Table 3 | Oligonucleotide sequences

RT-qPCR		Oligonucleotides (5' to 3')	
**Gene**	**AGI code**	**Forward**	**Reverse**
UBQ5	AT3G62250	GGTGCTAAGAAGAGGAAGAAT	CTCCTTCTTCTGGTAAACGT
S16	AT5G18380 AT2G09990	TTTACGCCATCCGTCAGAGTAT	TCTGGTAACGAGAACGAGCAC
PR1	AT2G14610	GTGCCAAAGTGAGGTGTAACAA	CGTGTGTATGCATGATCACATC
PR2	AT3G57260	TGGTGTCAGATTCCGGTACA	CATCCCTGAACCTTCCTTGA
PR5	AT1G75040	ATCGGGAGATTGCAAATACG	GCGTAGCTATAGGCGTCAGG

The analyses were performed according to Bauer, S. et al. (2021). Normalization was based on *UBQ5* and *S16* transcripts (Vandesompele, J. et al. Genome Biology 3, research0034.1–0034.11 (2002).

UGT76B1 complemetation with fluorescently labeled fusion protein	Oligonucleotides (5' to 3')	
	**Sequence**	**Remarks**
UGT76B1pro_GW_f	GGGGACAAGTTTGTACAAAAAAGCAGGCTcggttaaacataaaccatgt	5'-forward primer to amplify *UGT76B1* promoter fragment with B1-GATEWAY extension
UGT76B1pro-mTFP-Hy_R	cctcgcccttgctcaccatttttgttgtgaatttctctc	3'-reverse primer to amplify *UGT76B1* promoter with mTFP overlap
UGT76B1pro-mTFP-Hy_F	gagagaaattcacaacaaaa**ATG**gtgagcaagggcgagg	5`-forward primer to amplify mTFP from pNIGEL19 (Geldner et al., 2009) with *UGT76B1* $_{pro}$ overlap
mTFP-UGT76B1utr-Hy_R	gttttgtttctctagtctccattcctgcaccttgtacagctcgtccatgc	3'-reverse primer to amplify mTFP with linker and *UGT76B1* ATG start codon overlap
mTFP-UGT76B1utr-Hy_F	ctgtacaagggtgcagga**ATG**gagactagagaaacaaaacca	5'-primer to amplify the coding region of the *UGT76B1* gene with linker and overlap to mTFP
UGT76B1gene_GW_R	GGGGACCACTTTGTACAAGAAAGCTGGGTctgtgatttctgctttctgat	3'-primer to amplify the *UGT76B1* gene with B2 GATEWAY extension

Tissue-specific knockout	Oligonucleotides (5' to 3')	
	**Forward**	**Reverse**
TSKO-promoter-CO2	TTGAAGACATGGAGTAGCTTTGCTTTTTTCTCTATTAAG	TTGAAGACATATGGTATCGTTATTAACTAGGGTTCTTGA
TSKO-promoter-CASP1	TTGAAGACATGGAGTTAAATGTGCATAAAAGTGAGTATG	TTGAAGACATATGGTTTCTCTTGCAATTGGGG
TSKO-sgRNA-mTFP	attGCTCCCAAGAGTAGCCCTCG	aaacCGAGGGCTACTCTTGGGAGC
TSKO-sgRNA-UGT76B1	attgTTGGAGAGTTGAACTCAGTG	aaacCACTGAGTTCAACTGTCCAA

Supplementary Table. 4 | Microbe strain number and source of origin

Species	Strain Designations	Source
Alternaria alternata	IMB 12090	DSMZ-German Collection of Microorganisms and Cell Cultures GmbH
Alternaria brassicicola	529	Günther Bahnweg, Helmholtz Zentrum München, Germany
Aspergillus niger	Thor 2	DSMZ-German Collection of Microorganisms and Cell Cultures GmbH
Botrytis cinerea	310	DSMZ-German Collection of Microorganisms and Cell Cultures GmbH
Colletotrichum incanum	238704	Stéphane Hacquard, MPI für Züchtungsforschung Köln, Germany
Colletotrichum tofieldiae	Ct0861	Stéphane Hacquard, MPI für Züchtungsforschung Köln, Germany
Fusarium culmorum	IMB 12346	DSMZ-German Collection of Microorganisms and Cell Cultures GmbH
Fusarium graminearum	FR 26	DSMZ-German Collection of Microorganisms and Cell Cultures GmbH
Fusarium oxysporum	47	Christian Steinberg, INRAe UMR agroecologie, France
Fusarium oxysporum	5176	Günther Bahnweg, Helmholtz Zentrum München, Germany
Laccaria bicolor	S238N	INRAe-Nancy, France
Meliniomyces bicolor	L9172K	University Tartu, Estonia
Mucor circinelloides	p8d0-3	J. Philipp Benz, Technical University München, Germany
Penicillium pinophilum	1960	DSMZ-German Collection of Microorganisms and Cell Cultures GmbH
Phytophthora parasitica	INRA-310	Agnès Attard, Institut Sophia Agrobiotech, Sophia Antipolis, France
Sclerotinia scerotiorum	537	Günther Bahnweg, Helmholtz Zentrum München, Germany
Serendipita indica	DSM 11827	Wolfgang Dröge-Laser, Julius-Maximilians-Universität Würzburg, Germany
Trichoderma hamatum	QL15d1	Monika Schmoll, Austrian Institute of Technology GmbG, Austria
Trichoderma harzianum	ES891	Monika Schmoll, Austrian Institute of Technology GmbG, Austria
Trichoderma velutinum	GL1561	Monika Schmoll, Austrian Institute of Technology GmbG, Austria
Trichoderma reesei	QM6a	Monika Schmoll, Austrian Institute of Technology GmbG, Austria
Ustilago nuda	265	Günther Bahnweg, Helmholtz Zentrum München, Germany
Verticilium albo-atrum	334	Günther Bahnweg, Helmholtz Zentrum München, Germany
Verticilium longisporum	Vl43	Wolfgang Dröge-Laser, Julius-Maximilians-Universität Würzburg, Germany
